# N-glycosylation of cervicovaginal fluid reflects microbial community, immune activity, and pregnancy status

**DOI:** 10.1038/s41598-022-20608-7

**Published:** 2022-10-10

**Authors:** Gang Wu, Paola Grassi, David A. MacIntyre, Belen Gimeno Molina, Lynne Sykes, Samit Kundu, Cheng-Te Hsiao, Kay-Hooi Khoo, Phillip R. Bennett, Anne Dell, Stuart M. Haslam

**Affiliations:** 1 Department of Life Sciences, Imperial College London, London, UK; 2 March of Dimes Prematurity Research Centre at Imperial College London, London, UK; 3 Institute of Reproductive and Developmental Biology, Imperial College London, Hammersmith Hospital Campus, Du Cane Road, London, UK; 4 The Parasol Foundation Centre for Women’s Health and Cancer Research, St Mary’s Hospital, London, W1 2NY UK; 5 Institute of Biological Chemistry, Academia Sinica, Taipei, Taiwan

**Keywords:** Mass spectrometry, Glycobiology, Glycomics

## Abstract

Human cervicovaginal fluid (CVF) is a complex, functionally important and glycan rich biological fluid, fundamental in mediating physiological events associated with reproductive health. Using a comprehensive glycomic strategy we reveal an extremely rich and complex N-glycome in CVF of pregnant and non-pregnant women, abundant in paucimannose and high mannose glycans, complex glycans with 2–4 N-Acetyllactosamine (LacNAc) antennae, and Poly-LacNAc glycans decorated with fucosylation and sialylation. N-glycosylation profiles were observed to differ in relation to pregnancy status, microbial composition, immune activation, and pregnancy outcome. Compared to CVF from women experiencing term birth, CVF from women who subsequently experienced preterm birth showed lower sialylation, which correlated to the presence of a diverse microbiome, and higher fucosylation, which correlated positively to pro-inflammatory cytokine concentration. This study is the first step towards better understanding the role of cervicovaginal glycans in reproductive health, their contribution to the mechanism of microbial driven preterm birth, and their potential for preventative therapy.

## Introduction

All cells have a sugar “coat” consisting of diverse glycans, which function as primary molecules for cellular recognition, adhesion, signalling and host–pathogen/host commensal interactions^1–4^. Human N-glycan biosynthesis starts with a precursor structure, from which high mannose, hybrid and complex type glycans are synthesized^5^. The antennae on complex and hybrid glycans carry important sequences, such as Lewis antigens, Sialyl Lewis antigens, ABO blood groups and poly LacNAc, which are recognised by glycan binding proteins (GBP), which are also known as lectins^6,7^. The glycans of glycoproteins expressed along the human female reproductive tract are of fundamental importance at all stages of human pregnancy, from conception and implantation to delivery^2^. In addition to acting as potential ligands for GBP of the host immune system, they can serve as adhesion and attachment points for both pathogenic and commensal microbes. Bacterial vaginosis (BV) is a polymicrobial imbalance of the vaginal microbiota, which is associated with development of precancerous cervical lesions, pelvic inflammatory disease, endometritis, tubal infertility and preterm birth^8–11^. The BV-associated bacteria *Gardnerella vaginalis* and *Prevotella bivia* produce extracellular sialidases to hydrolyse mucosal sialoglycans and transport the released sialic acid inside the bacterium cells for catabolism^12,13^, which may benefit their survival under some nutrient deprived conditions. Recent proteome-wide prediction studies of bacteria have identified > 100,000 putative bacterial GBP^14,15^. Genome screening found that vaginal bacterial species associated with infection and inflammation produce a larger number of GBP than commensals, potentially allowing them to bind a wider range of glycans in the vagina^14^. Moreover, the number of predicted bacterial lectins and their specificities correlated with pathogenicity. Secretor status, related to the expression of the *FUT2* gene, is responsible for the synthesis of the H-antigen glycan structure in the mucus and also provides the precursor for mucosal blood group A and B synthesis. Approximately 20% of the human population presents a mutation in *FUT2*, which abolishes α(1,2)fucosyltransferase activity. This ‘nonsecretor’ phenotype is associated with a 2.4 to 4.4 fold increased relative risk for recurrent vaginitis by *C. albicans*^16^, and maternal lack of H-antigen production was found to be a risk factor for preterm birth^17^. Moreover, non-secretors with *Lactobacillus* depleted vaginal microbiota in early pregnancy have significantly shorter pregnancies compared to women with *Lactobacillus* dominated composition, a relationship not seen in women who are secretors^18^.

Human physiological parturition is a pro-inflammatory process^19^. Histological studies have found leukocytes, especially macrophages and neutrophils, infiltrate the human myometrium, fetal membrane and the cervix, around the time of parturition^20,21^, which is accompanied by increased levels of pro-inflammatory cytokines, such as IL-1β, IL-6, IL-8 and TNF-α. Preterm labour is associated with early activation of inflammation at the cervical-vaginal and maternal–fetal interface, and is commonly thought to be due to the presence of pathogenic microbes^22,23^. Neutrophils are the key immune cells recruited to fetal membranes as a result of microbial driven inflammation^24^, and are likely to be key immune mediators at the cervical-vaginal interface^25^. Infection is thought to contribute to at least one third of preterm birth cases^9^, and there is mounting evidence for the role of host-vaginal microbial interactions in these processes^9,23,26,27^. The cervix functions as both a mechanical and immunological barrier to ascending infection during pregnancy. This is in part mediated by cervical secretion of highly glycosylated glycoproteins, which are a major constituent of cervical mucus^28,29^. It is estimated that up to 80% of the weight of cervical mucins can be attributed to glycans^30,31^. Cervicovaginal fluid (CVF) thus represents a heterogeneous mixture of endocervical and vaginal secretions, as well as epithelial cells, leukocytes and antibodies, all of which are highly decorated by complex glycan structures. In other mucosal niches, glycan mediation of immune response is well characterised and includes crosstalk between glycans and immune receptors that regulate leukocyte migration, antigen presentation, immune activation and antibody responses^1,32–34^.

Proteomic analyses of CVF have identified more than 1200 proteins in the CVF^35^, many of which can be functionally associated with immune responses, infection, and pregnancy complications including biochemical processes involved in the onset of preterm labour^27,36–45^. Recently a desorption electrospray ionization mass spectrometry (DESI-MS) based study, which allows ionisation of mucosal biomass directly from a standard clinical swab, demonstrated changes in the mucosal metabolome associated with pregnancy, microbiota composition and immune activation that associates with preterm birth risk^46^.

Despite their importance in mediating various physiological events associated with reproductive health, particularly during pregnancy, to our knowledge, detailed profiling of CVF glycans has not been reported. In this study, we used a comprehensive glycomic strategy to characterize the N-glycans in the CVF of pregnant and non-pregnant women. We demonstrate that glycan structural features such as fucosylation and sialylation are associated with pregnancy status, microbial community state, immune activity, and pregnancy outcome.

## Results

### N-glycan structural profiling

CVF samples from a total of 10 donors with diverse ethnicities were collected for analysis. 4 of the donors were non-pregnant women, while 6 of the 10 donors were pregnant women defined at high risk of spontaneous preterm delivery (sPTB). Of these, 3 delivered at term, while 3 delivered preterm (< 37 weeks of pregnancy) (Table 1, Supplementary Table S1).

**Table 1:** Summary of CVF samples collected for glycomic analysis.

Sample code	Pregnancy outcome	CST	Ethnicity	Gestation (weeks + days)	ABO blood group
NP1	Non-pregnant	Not available	Caucasian	0 + 0	O
NP2	Non-pregnant	Not available	South Asian	0 + 0	A
NP3	Non-pregnant	Not available	African	0 + 0	A
NP4	Non-pregnant	Not available	Caucasian	0 + 0	A
P1	Term	I	Caucasian	39 + 0	O
P2	Term	I	African	39 + 3	B
P3	Term	IV-B	African	39 + 3	O
P4	Preterm	I	South Asian	27 + 3	B
P5	Preterm	IV-B	African	31 + 3	B
P6	Preterm	IV-B	South Asian	22 + 4	B

**Figure 1:**
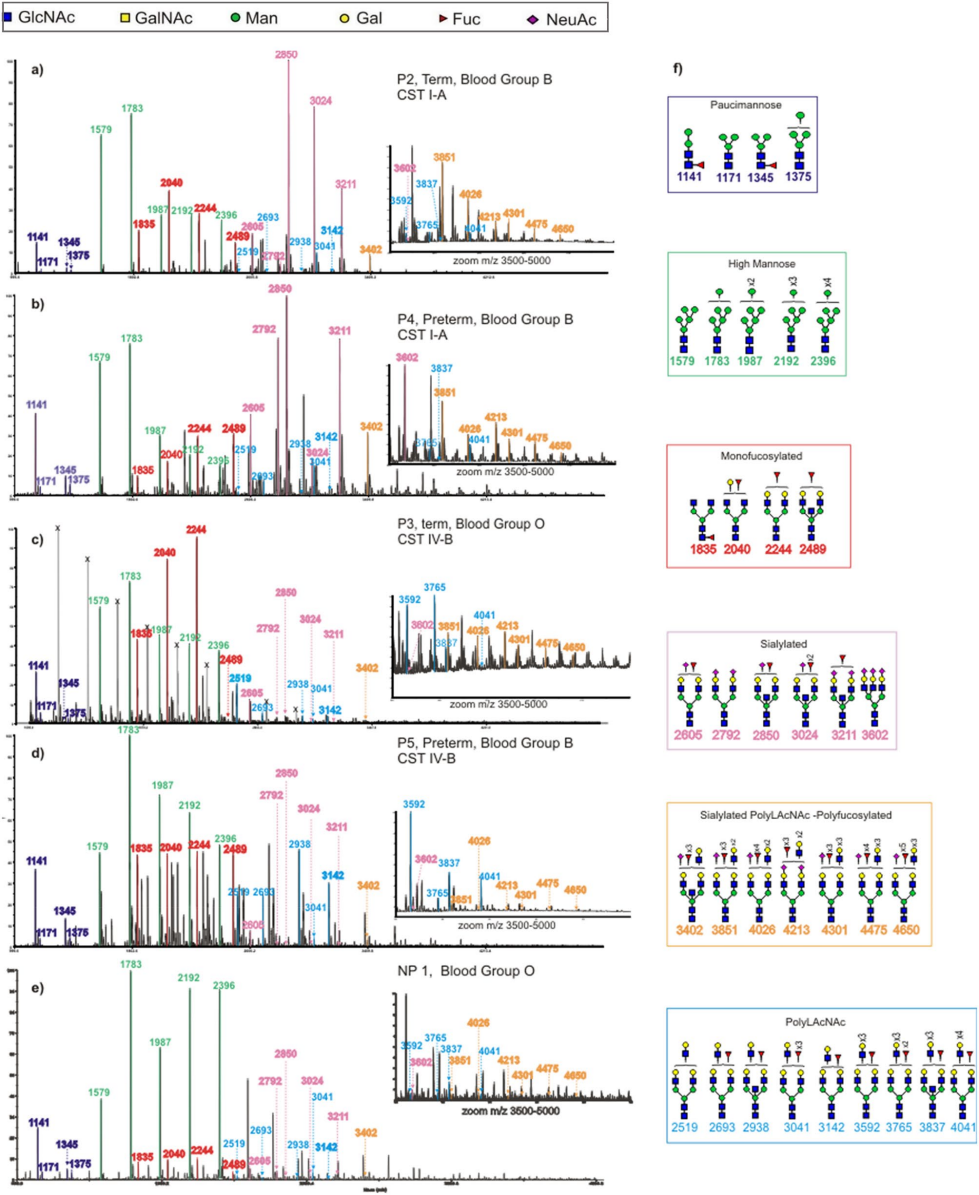
MALDI-TOF mass spectra (m/z 1000–5000) of N-glycans isolated from CVF of a donor with CST I-A who delivered at term in panel (**a**), a donor with CST I-A who delivered preterm in panel (**b**), a donor with CST IV-B who delivered at term in panel (**c**), a donor with CST IV-B who delivered preterm in panel (**d**) and a non-pregnant donor in panel (**e**). Donors in panel (**a**), (**b**) and (**d**) are blood group B, while donors in panels (**c**) and (**e**) are blood group O. The N-glycans from CVF were released by PNGase F and permethylated prior to MALDI-TOF and TOF-TOF profiling. Each spectrum is shown in a single panel, and all data are normalized to the most abundant component, which is designated as 100%. For clarity, a zoomed in panel is inserted for masses above 3500; colour coding has been used to distinguish families of glycans: paucimannose N-glycans are flagged as dark purple, peaks in green show high-mannose N-glycans, peaks in magenta are complex biantennary monofucosylated glycans, peaks in pink are sialylated complex N-glycans, peaks in orange are large PolyLacNAc structures decorated with multiple sialic acid and fucose residues, and in light blue are PolyLacNAc glycans decorated with 0–3 fucose residues. Peaks in light grey marked with X are known polyhexose contaminants. Structures of the colour coded peaks are represented in the corresponding coloured rectangle in panel (**f**). Main structures are depicted. Assignments are based on composition, tandem MS and knowledge of biosynthetic pathways. All molecular ions are [M + Na]^+^. Residues above a bracket have not had their location unequivocally defined.

A detailed MALDI-MS based glycomic characterisation of permethylated N-glycans was undertaken. This produces [M + Na]^+^ molecular ions. An overall relative quantitation of paucimannose, high mannose, hybrid and complex glycans is shown in Supplementary Fig. S1. More detailed structural characterization is illustrated in Fig. 1. Paucimannose gycans were identified in the low mass range at m/z 1141, 1171, 1345 and 1375, (Man_2-4_GlcNAc_2_Fuc_0-1_). The glycans in the mid mass range were dominated by high mannose glycans (m/z 1579–2396, Man_5-9_GlcNAc_2_) and bi-antennary complex glycans with fucosylation and/or sialylation (m/z 1835–3211, NeuAc_0-2_Gal_0-2_Man_3_GlcNAc_4-5_Fuc_0-1_). The glycans at higher mass range contained multiple LacNAc units (Gal-GlcNAc) modified by different numbers of fucose and sialic acid (m/z 3402–4650, NeuAc_0-3_Gal_3-6_Man_3_GlcNAc_4-8_Fuc_0-5_). The largest complex N-glycan observed was at m/z 6259 corresponding to a composition of Gal_9_Man_3_GlcNAc_11_Fuc_6_ (Supplementary Table S2). More detailed N-glycan structural analysis was achieved by MS/MS analysis of selected molecular ions. This proved that the mono-fucosylated glycans can be both core or antenna fucosylated (Supplementary Fig. S2a), and that peaks with composition consistent with multiple LacNAc units are a mixture of structural isomers with varying numbers and lengths of antennae. Complex glycans with 2–4 LacNAc units were identified in the medium mass range and glycans with poly LacNAc units in the high mass range (Fig. 1). Glycans above m/z 5000 included high numbers of LacNAc units exhibiting diverse modifications by Fuc and NeuAc (Supplementary table S2).

The MS/MS analysis reveals that structures with extended antennae are often favoured over branched structures (Supplementary Fig. S2b). These extended antennae were decorated by several fucose residues producing diverse poly Lewis antigens. LacdiNAc (GalNAcβ1-4GlcNAc) containing structures were identified at *m*/*z* 2285, 2326, 2500 and 2674, which were supported by MS/MS fragmentation analysis (Supplementary Fig. S2c). Interestingly, sample P5, from a donor with CST IV-B who delivered preterm at 31 + 3 weeks, showed a distinct pattern of glycan abundances compared with other samples. Its N-glycome was dominated by non-sialylated, and non-/mono-fucosylated glycans (Supplementary Fig. S3). In addition, this sample had high levels of N-glycans with truncated antennae terminated by GlcNAc.

A distinct N-glycan composition was also observed in CVF from non-pregnant donors (Fig. 1e, Supplementary Fig. S4). Compared to pregnant women, spectra obtained from non-pregnant donors are dominated in the lower mass range by high mannose N-glycans (m/z 1579–2396, Man_5-9_GlcNAc_2_), while the mid mass range is dominated by biantennary complex poly-fucosylated structures at m/z 2592, 2766 and 2940 (Gal_2_Man_3_GlcNAc_4_Fuc_3-5_). The higher mass range is dominated by large polyLacNAc, heavily fucosylated glycans such as structures at m/z 3215, 3563, 3737, 4012, 4186, 4361, 4810 and 4984 (Gal_2-5_Man_3_GlcNAc_4-7_Fuc_3-9_). Complex biantennary, triantennary and tetrantennary glycans capped by one to four sialic acids, were also detected, (eg., m/z 2605, 2966, 3211, 3776, 4124, 4473, 4761) but at lower abundance than exclusively fucosylated glycans.

**Figure 2:**
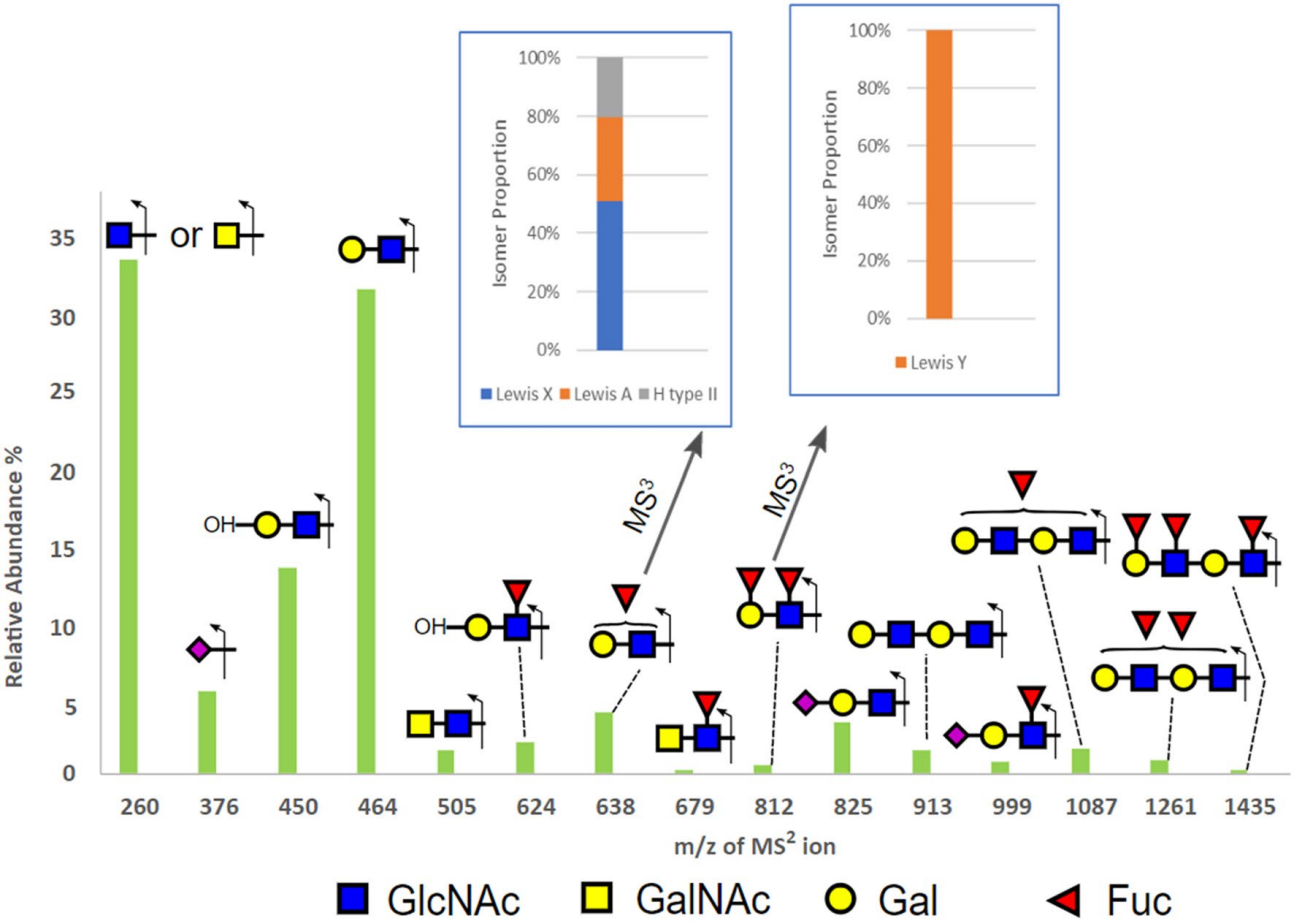
Glycotope centric analysis of N-glycans from sample P3. All N-glycans were selected for CID fragmentation to produce MS2 ions. The summed intensity for each of the detected diagnostic MS2 ions normalized to their total was plotted to provide an overall assessment of the relative abundance of various glycotopes. The glycotopes represented by MS2 ions at m/z 638 and m/z 812 were further selected for additional stage of fragmentation (MS3) to identify their respective isomeric constituents, as shown in the insets.

The complexity of CVF N-glycans with their multiple fucosylation and sialylation led us to apply additional complementary analytical strategies to facilitate deeper characterization of CVF N-glycan structures. Glyco-epitope centric N-glycan analysis using an Orbitrap mass spectrometer confirmed the presence of diverse glycotopes in a selected sample P3 (Fig. 2). MS^2^ fragments of N-glycans detected LacdiNAc structures at m/z 505 and 679, terminal Lewis antigens at m/z 638 and 812, internal Lewis antigens at m/z 624, and Sialyl Lewis antigens at m/z 999. In addition, poly Lewis antigens were observed at m/z 1261 and m/z 1435. The MS^2^ data also showed terminal GlcNAc or GalNAc at m/z 260, predicted to arise from bisecting glycans, glycans with truncated antennae, or LacdiNAc structures. The MS^2^ peak at m/z 812 was selected for further fragmentation to obtain MS^3^ data, which mainly detected the Lewis Y antigen. However, the MS^3^ analysis of the MS^2^ peak at m/z 638 revealed a mixture of Lewis X, Lewis A and the blood group H antigen, again highlighting the structural complexity of CVF N-glycans.

### Correlation of fucosylation and sialylation to pregnancy status

**Figure 3:**
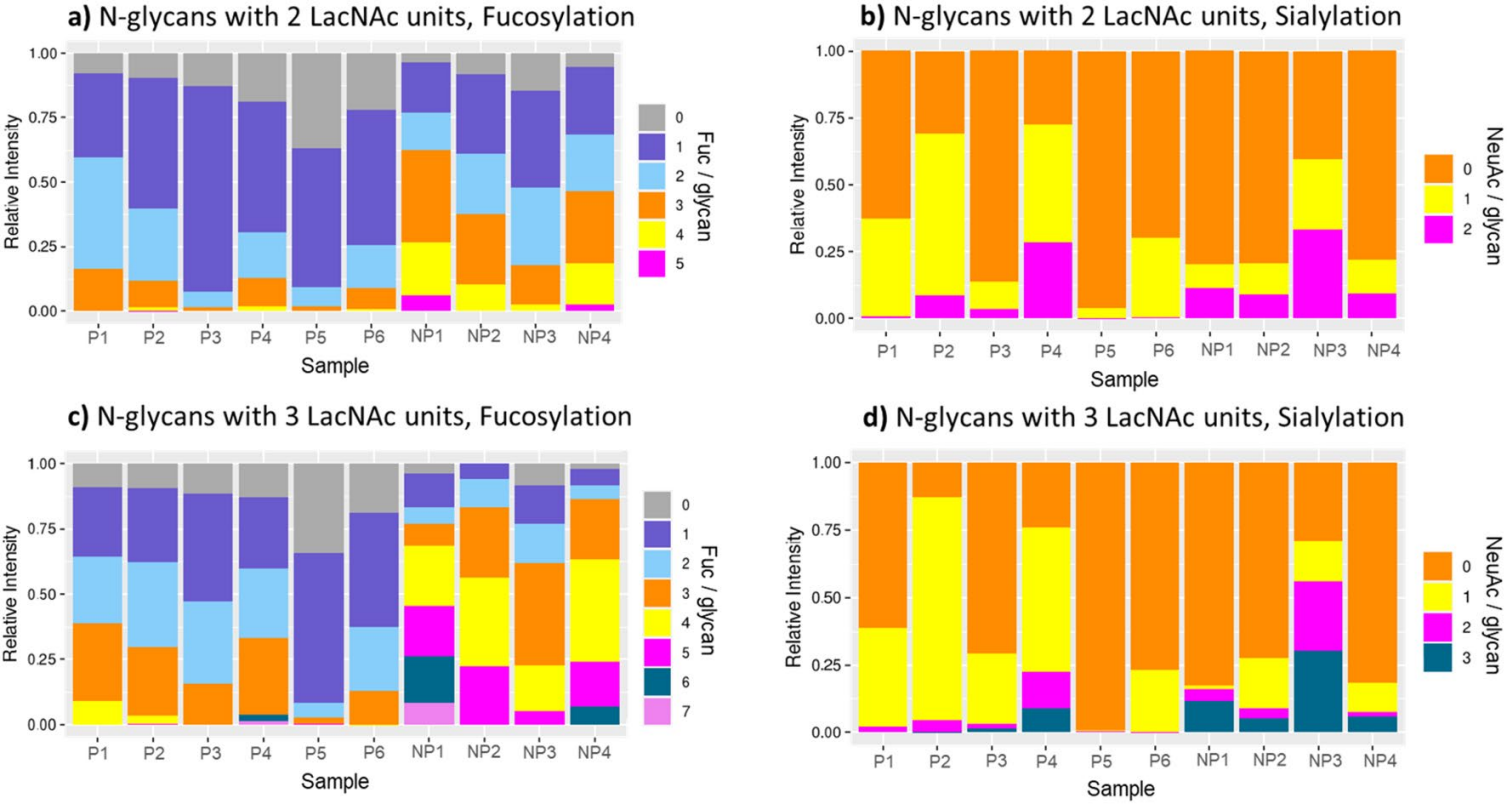
Overall view of fucosylation and sialylation levels of N-glycans with 2 LacNAc units (**a**,**b**) and 3 LacNAc units (**c**,**d**). The glycans were subgrouped according to the number of Fuc or NeuAc they have. The summed intensity of glycans in a subgroup divided by the total glycan intensity was calculated as a relative intensity of the subgroup.

We next focused on quantitative analysis of the fucosylation and sialylation of the CVF N-glycans containing 2 or 3 LacNAc units (Fig. 3). Glycans with 2 LacNAc units were shown to have up to 5 Fuc/glycan (Fig. 3a) whereas glycans with 3 LacNAc units had up to 7 Fuc/glycan (Fig. 3c). In terms of sialylation, glycans with 2 LacNAc units had up to 2 sialic acids (Fig. 3b) and glycans with 3 LacNAc units had up to 3 sialic acids (Fig. 3d). Variations of fucosylation and sialylation were observed among the samples. Quantitative analysis of Sample P5 showed extremely low levels of poly-fucosylation and sialylation for glycans with 2 or 3 LacNAc units (Fig. 3).

**Figure 4:**
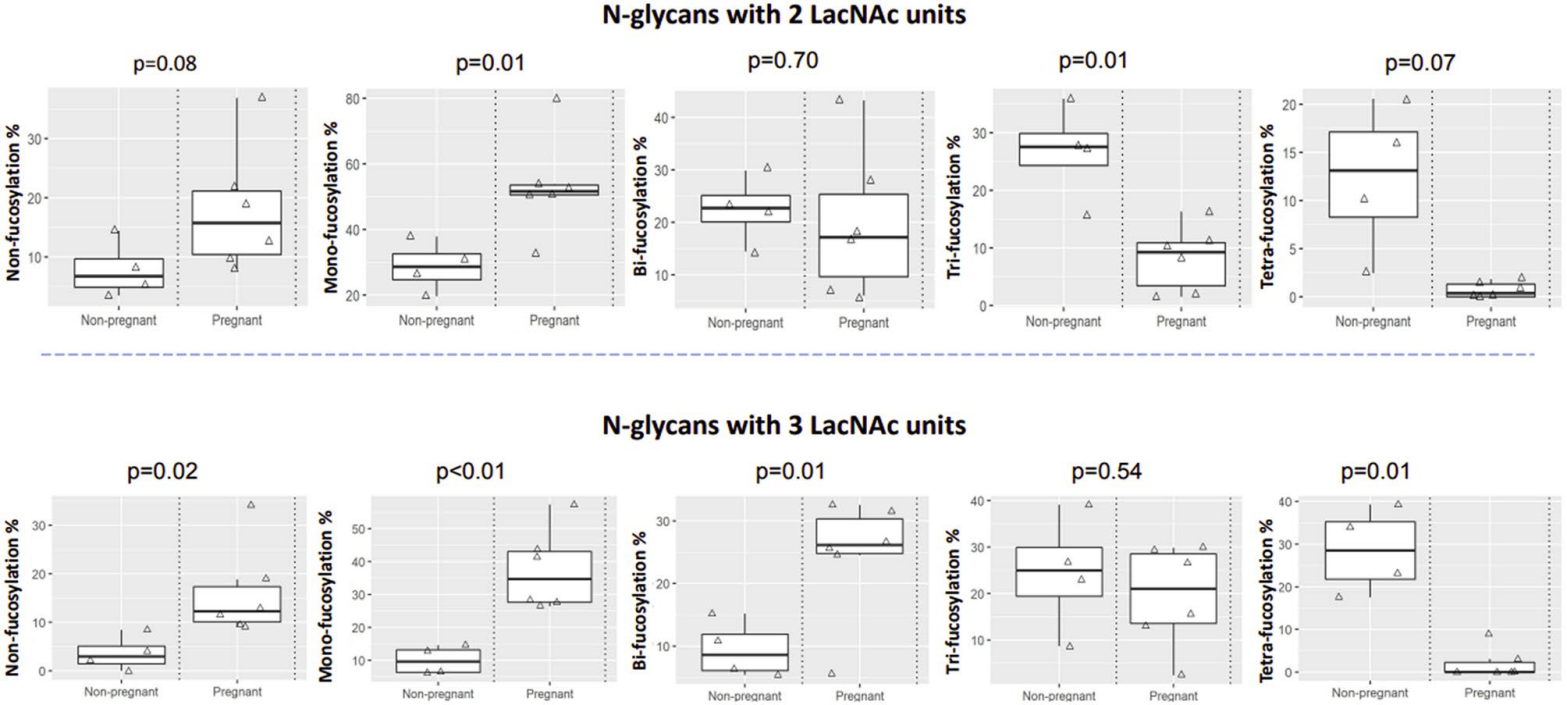
Fucosylation variations between non-pregnant and pregnant samples. N-glycans with 2 LacNAc units (top panel) and 3 LacNAc units (bottom panel) were selected for analysis. The proportion of summed intensity of glycans with non-fucosylation, mono-fucosylation, bi-fucosylation, tri-fucosylation and tetra-fucosylation to total glycan intensity was calculated for data analysis.

**Figure 5:**
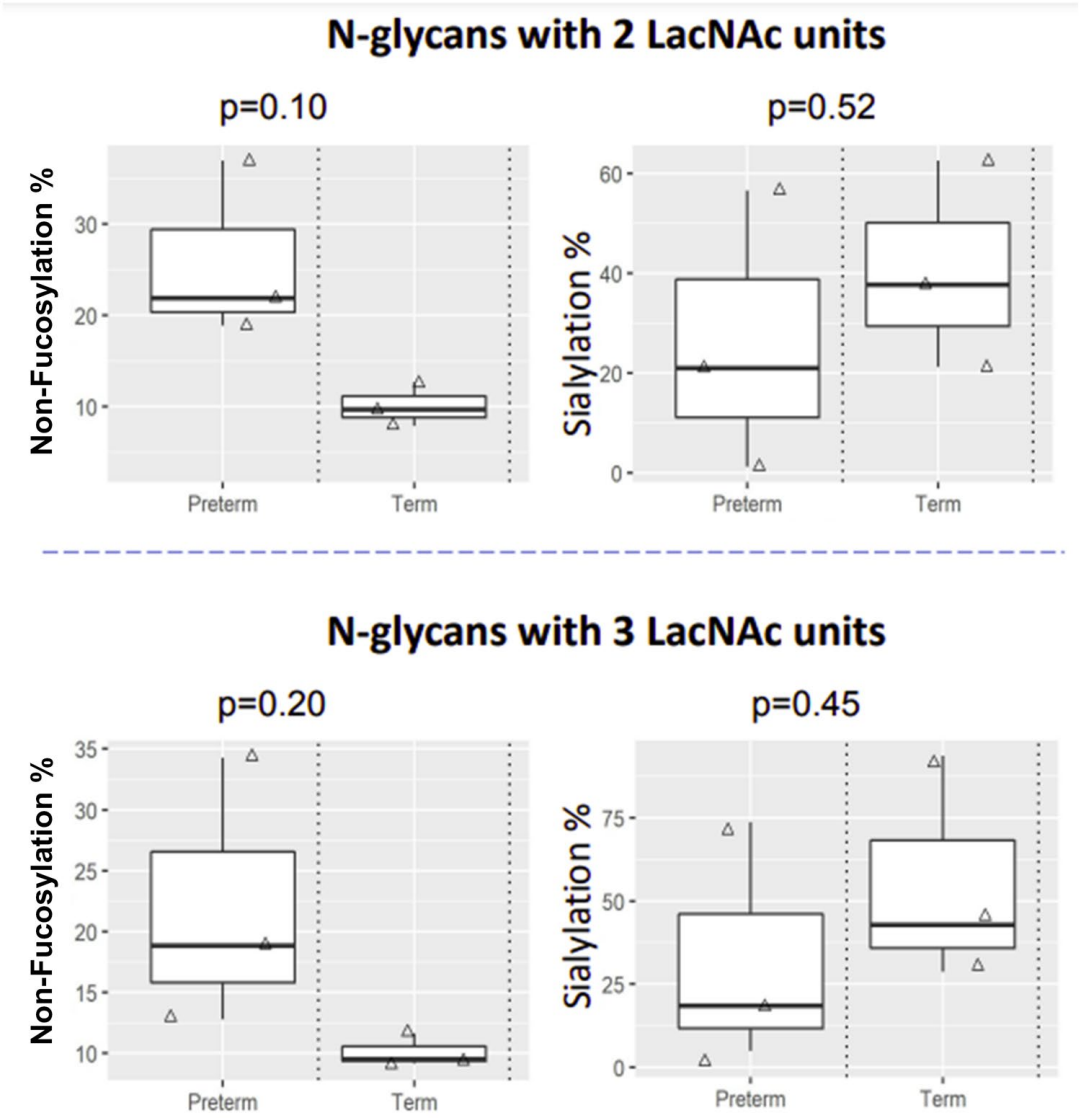
Fucosylation and sialylation variations between preterm and term samples. N-glycans with 2 LacNAc units (top panel) and 3 LacNAc units (bottom panel) from pregnant samples were selected for analysis. Non-fucosylation % was calculated as the relative intensity of non-fucosylated glycans to the intensity of all glycans. Sialylation % was calculated as the relative intensity of sialylated glycans to the intensity of all poly-fucosylated glycans.

As shown in Fig. 4, differential fucosylation was observed between non-pregnant and pregnant samples. For glycans with 2 LacNAc units, the pregnant samples had higher levels of non-fucosylated glycans and mono-fucosylated glycans. However, this trend started to reverse for bi-fucosylated glycans and was completely reversed for tri- and tetra-fucosylated glycans. Similar results were observed for glycans with 3 LacNAc units, except that the reversing point was at the tri-fucosylated glycans. The proportion of tetra-fucosylated glycans decreased to almost zero in the pregnant samples, but was retained at about 12% for glycans with 2 LacNAc units and 30% for glycans with 3 LacNAc units in the non-pregnant samples. An initial trend of fucosylation and sialylation changes were detected between preterm and term samples (Fig. 5). CVF samples of women who delivered preterm showed higher levels of non-fucosylated glycans among glycans with 2 or 3 LacNAc units. The sialylation level among poly-fucosylated glycans tended to be lower for the preterm samples.

### Correlation of fucosylation and sialylation to microbial composition

**Figure 6:**
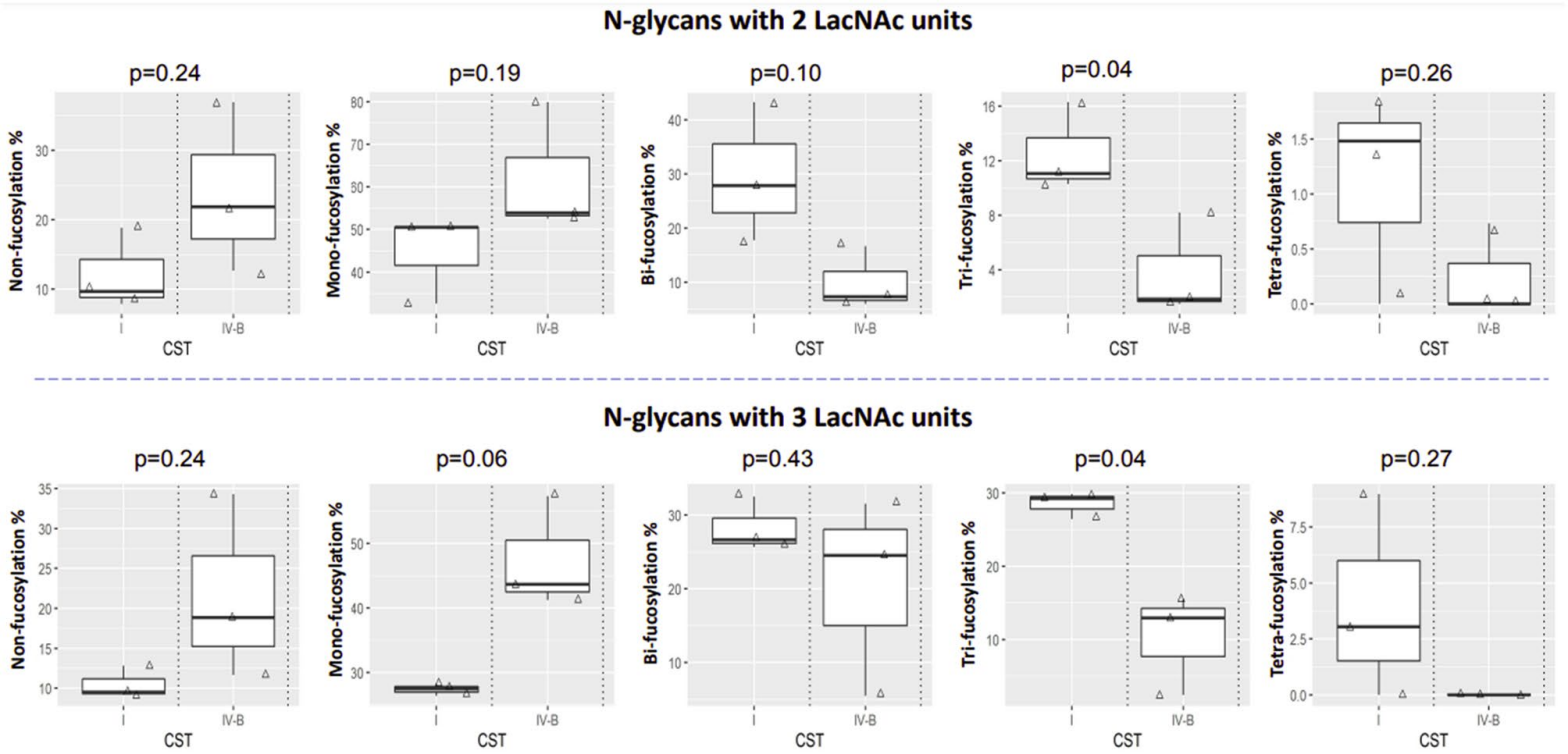
Correlation of CST to fucosylation depended on the number of Fuc per glycan. N-glycans with 2 LacNAc units (top panel) and 3 LacNAc units (bottom panel) were selected for analysis. The proportion of summed intensity of glycans with non-fucosylation, mono-fucosylation, bi-fucosylation, tri-fucosylation and tetra-fucosylation to total glycan intensity was calculated.

**Figure 7:**
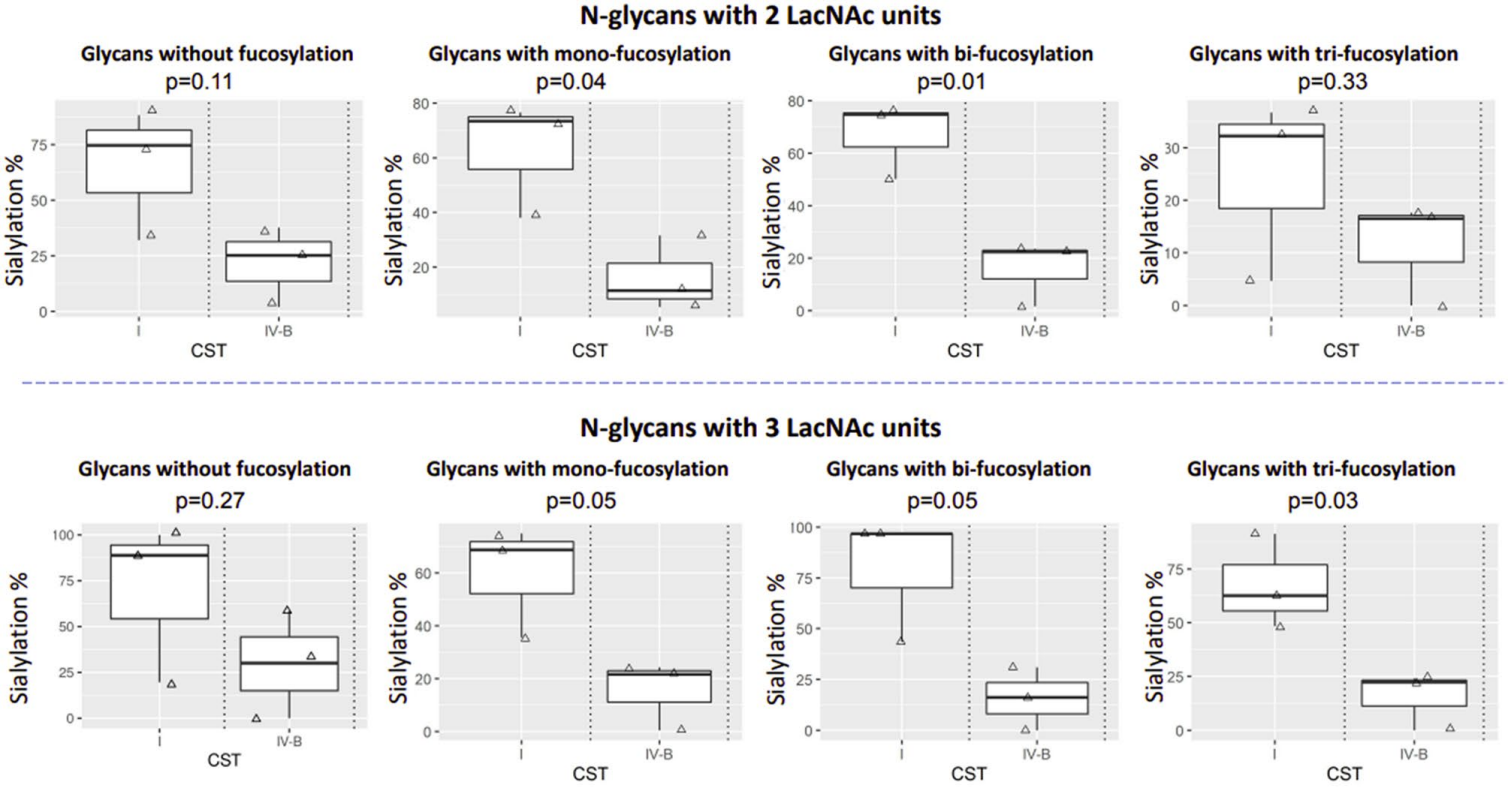
CVF samples with CST IV-B had lower levels of sialylation than those with CST I, regardless of fucosylation. N-glycans with 2 LacNAc units (top panel) and 3 LacNAc units (bottom panel) were selected for analysis. The glycans were grouped based on the number of Fuc they have. In each group, the relative intensity of sialylated glycans to total glycan itensity was calculated as sialylation %.

Community State Type (CST) was used to characterise the vaginal microbial community status. Analysis of vaginal microbiota revealed that 3 of the pregnant samples had a vaginal bacterial community dominated by *L*. *crispatus*, consistent with Community State Type I (CST I) as described elsewhere^47^, while 3 had a CST IV-B, characterised by *G. vaginalis* and *A. vaginae* dominance. Of the CST I samples two (P2, P4) were CST I-A (almost completely dominated by *L. crispatus*), and one (P1) was CST I-B (mostly dominated by *L. crispatus* but also containing low abundance of *L. gasseri*, *L. jensenii* and *G. vaginalis*). Of the samples that were classified as CST IV-B, there was a high relative abundance of *G. vaginalis* in two (P5 and P6), and a moderate relative abundance of *A. vaginae* and *L. acidophilus* in the other (P3) (Supplementary Fig. S5). The correlation between fucosylation and vaginal CST was investigated in relation to the number of Fuc residues per glycan (Fig. 6). N-glycans with 2 LacNAc units and those with 3 LacNAc units showed the same trend: samples from CST IV-B donors had higher levels of non-fucosylated glycans and mono-fucosylated glycans, but lower levels of bi-fucosylated, tri-fucosylated and tetra-fucosylated glycans, compared with CST I-A/B donors. Detailed analysis of the correlation between sialylation and CST is shown in Fig. 7. Sialylation of glycans with different numbers of Fuc was analysed separately. The same trend was found regardless of the fucosylation status: CST IV-B samples consistently showed a lower level of sialylation compared to CST I-A/B samples. Our results suggest that the presence of a high relative abundance of Lactobacillus spp. is associated with a higher percentage of sialylation, bi-, tri- and tetra-fucosylated glycans, and lower levels of non- or mono-fucosylated glycans.

### Correlation of fucosylation and sialylation to pro-inflammatory cytokines

**Figure 8:**
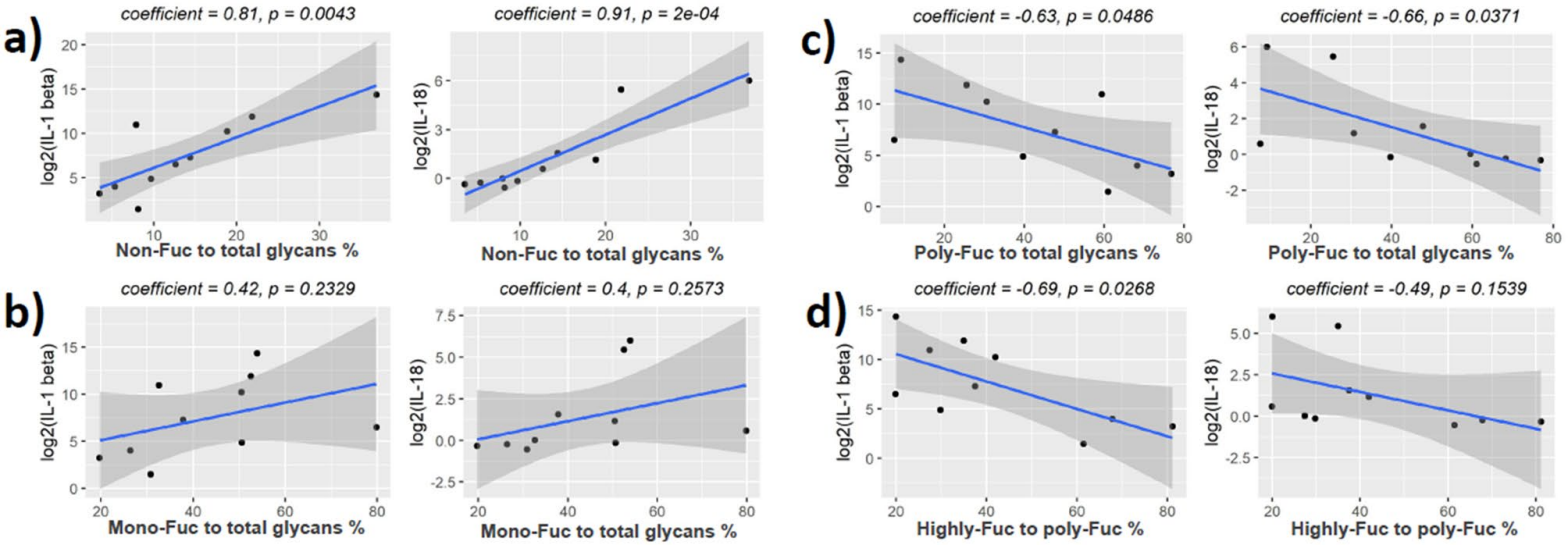
Correlation of fucosylation to IL-1 beta and IL-18 for glycans with 2 LacNAc units. Pearson's product-moment correlation was used for statistical analysis. (**a**) Relative intensity of non-fucosylated glycans to the intensity of all glycans. (**b**) Relative intensity of mono-fucosylated glycans to the intensity of all glycans. (**c**) Relative intensity of poly-fucosylated glycans to the intensity of all glycans. (**d**) Relative intensity of highly fucosylated glycans to the intensity of poly-fucosylated glycans. Highly fucosylated glycans were defined as those with at least 3 Fuc per glycan. Poly-fucosylated glycans were defined as those with at least 2 Fuc per glycan.

**Figure 9:**
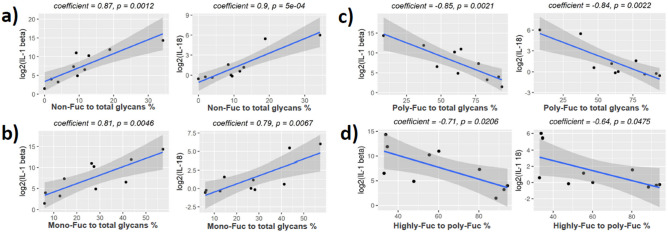
Correlation of fucosylation to IL-1 beta and IL-18 for glycans with 3 LacNAc units. Pearson's product-moment correlation was used for statistical analysis. (**a**) Relative intensity of non-fucosylated glycans to the intensity of all glycans. (**b**) Relative intensity of mono-fucosylated glycans to the intensity of all glycans. (**c**) Relative intensity of poly-fucosylated glycans to the intensity of all glycans. (**d**) Relative intensity of highly fucosylated glycans to the intensity of poly-fucosylated glycans. Highly fucosylated glycans were defined as those with at least 4 Fuc per glycan. Poly-fucosylated glycans were defined as those with at least 2 Fuc per glycan.

Strong correlation was found when fucosylation was mapped to levels of IL-1β and IL-18 (Figs. 8 and 9). The proportion of non-fucosylated glycans (Fig. 8a) and the proportion of mono-fucosylated glycans (Fig. 8b) to total glycans were positively correlated with cytokine concentration, while the proportion of poly-fucosylated glycans to total glycans (Fig. 8c) and the proportion of highly-fucosylated glycans to poly-fucosylated glycans (Fig. 8d) were negatively correlated with the cytokine concentrations. Similar correlations were observed for glycans with 3 LacNAc units (Fig. 9). No statistically significant correlations were seen with IL-8 and IL-6, however similar trends were observed (Supplementary Figs. S6 and S7). We also investigated levels of glycan sialylation and pro-inflammatory cytokines, however, only weak correlations were observed (Supplementary Figs. S1–S11).

## Discussion

Despite the importance of human CVF in shaping microbiota-host responses in the lower reproductive tract, to our knowledge, a detailed profiling of CVF N-glycans has not been reported. Our study characterises CVF as a glycan-rich environment with distinct and different N-glycosylation in relation to pregnancy status, microbial composition, immune activation, and pregnancy outcome. Our findings are based on a small cohort at present. This is due to a combination of factors including the collection device being more invasive than a simple swab and the labour intensive and high cost of sample processing. However, some initial trends have already been consistently observed. We also acknowledge that factors such as the stage of menstrual cycle or pregnancy, age, cervical length, blood group and ethnicity could influence the CVF glycosylation profile. We aim to study these factors with larger cohorts. The N-glycome of CVF is highly complex and abundant in paucimannose glycans and high mannose glycans in the low mass range. Complex glycans with 2–4 antennae in the medium mass range and glycans with poly LacNAc units in the high mass range are readily detectable. Antennae structures of detected N-glycans included terminal fucosylation of mono-fucosylated glycans, glycans with LacdiNAc structures, as well as sialylation and fucosylation patterns of extended poly LacNAc structures. Identification of a higher prevalence of glycan isomers carrying extended poly LacNAc chains compared to multi-antennary structures, suggests a biological role of the elongated antennae protruding from the mucin protein backbone^49,50^. While extended poly-*N*-acetyllactosamine glycans can serve as spacers and carriers of additional glycotopes through their modification with fucose and sialic acid, poly LacNAc itself has been identified as a recognition motif of galectins^51^, an important class of mammalian GBP involved in regulations of immune cell activities and microbial recognition as part of the innate immune system^52–54^. N-Glycan structures carrying extended poly LacNAc chains have recently been reported as a characteristic feature of neutrophil granules^55^, which suggests these structures in CVF might partially be due to leukocyte infiltration. High mannose glycans can be recognized by DC-SIGN, the mannose receptor and other mannose binding GBP, which have been found to be involved in inflammation and infection^56^.

Modifications of genital tract epithelia, mucus, and immune status occur throughout the menstrual cycle to accommodate conception and implantation^29^. At the time of conception, Sialyl Lewis X glycan epitopes on the human oocyte facilitates sperm-egg binding^57^, and glycans have also been implicated in the mediation of implantation^58^ and immune homeostasis at the maternal–fetal interface^59^. Once implantation is successful, the cervicovaginal interface undergoes further modification, with the primary aim of forming a cervical barrier to infectious microbes to protect the developing fetus. Our study shows that this physiological switch to a pregnancy phenotype also involves marked changes to cervicovaginal glycan profiles. In both non-pregnant and pregnant women, N-glycans were found to be a mixture of paucimannose and high-mannose structures in the lower mass range, biantennary complex poly-fucosylated structures in the mid mass range, and large PolyLacNAc glycans the higher mass range. However, spectra of non-pregnant women CVF are clearly dominated by high mannose glycans in the lower mass range, which could be a sign of reduced glycan maturation and differentiation^7^ or a different microbiota composition^25^ compared to pregnant women. Oligosaccharides carrying terminal mannose residues are the main host receptors for bacteria such as *E. coli*.^60^ A loss of high mannose residues on glycoproteins of the CVL from women with BV was observed by Wang et al*.*^61^, suggesting that native high mannose residues found on glycoproteins in fluids of the lower reproductive tract may act as natural inhibitors of pathogenic interactions, helping to prevent bacterial adhesion in women with normal microflora.

Glycans in the mid to high mass range of non-pregnant women show lower fucosylation and sialylation levels compared to pregnant women. This could be pointing again at a lower maturation and differentiation state of cervicovaginal proteins N-glycans and to a more quiescent immunological state which would allow easier transit of sperm and thus easier fertilisation.

We also demonstrated differences in glycan profiles between women who subsequently delivered preterm and women who delivered at term. The preterm samples showed higher levels of non-fucosylated glycans among glycans with 2 or 3 LacNAc units and lower sialylation among poly-fucosylated glycans. Desialylation of glycoproteins and glycolipids by sialidase leads to loss of or exposure of new glycan epitopes and interference with host immune recognition and binding of microbes^62^. It is feasible that this could contribute to the mechanism of microbial driven preterm birth. In support of this, high sialidase concentrations have been associated with late miscarriage and preterm delivery^63,64^. Further, we and others have reported an association between vaginal microbial composition and preterm birth risk^9^. High diversity vaginal microbial composition (referred to as community state type IV, CST IV), or dominance of the niche by *L. iners*, is associated with higher rates of preterm birth, whereas *L. crispatus* (CST I) dominance is protective^26,65,66^. In this study N-glycans with 2 and 3 LacNAc units isolated from CVF of women with CST IV have lower levels of both poly and highly-fucosylated glycans and sialyated glycans compared to women with CST-I.

Fucosylation and sialylation on LacNAc units can potentially produce different glycotopes, such as Lewis antigens, Sialyl Lewis antigens and poly Lewis antigens. Several studies have shown how glycans and their epitopes play a role in infective conditions such as HIV^67^ and *Candida albicans*^16^. *G. vaginalis* is one of the main bacteria associated with bacterial vaginosis and CST IV, which is characterised by depletion of *Lactobacillus* species and polymicrobial overgrowth of anaerobes^12,68^. BV is also associated with increased vaginal concentrations of glycosidases such as sialidase, α-galactosidase, β-galactosidase and α-glucosidase^69,70^ and decreased binding of lectins to both high mannose and α-2, 6 sialic acid. This observation suggests that bacteria sialidases and host sialoglycan receptors can be linkage specific, as also reported by other studies^1,71^. Findings from previous studies suggest that changes in glycosidases are accompanied by changes in glycosylation patterns in the vaginal fluid. On the other hand, a recent discovery found that Lactobacillus spp. Can create a protective micro-ecological environment through regulating the core fucosylation of vaginal epithelial cells^72^. Although glycosylation of the CVF could be influenced by hormone levels, it is thought that microbiota have a greater influence on CVF glycosylation than hormonal status^61^.

Similarly to CVF, the human gastrointestinal (GI) mucosa consists of extensive glycoproteins overlaying epithelial cells, interspersed by immune cells, which form a protective physical barrier, and serve as first line of defence against microorganisms and harmful substances^73,74^. Hydrolases, including exo- and endoglycosidases, are encoded in the genomes of mucin-degrading gut bacteria^73^ allowing them to metabolize sugars^73,75^, which are then exploited as nutrient source^76^. The mucins of the GI tract mucus layer also provide ligands for the attachment of bacteria^77^ and may facilitate invasion; moreover, mucus-binding proteins such as adhesins and GBP have been described in many lactic acid bacilli^78^. The human gut microbiome and its role in both health and disease has been extensively studied and its involvement in human metabolism, nutrition, physiology, and immune function is now well established^79–82^. It is highly probable that similar interactions exist in the mucosa of the female lower reproductive tract, creating a tight interconnection between glycans, microorganisms colonising the female reproductive tract and cervicovaginal immune homeostasis.

It is well established that local inflammation is a risk factor for preterm birth. However, only recently are we beginning to understand the relationship between inflammation, specific microbial communities, and preterm birth^21,23,46,83^. Here we observed that non-fucosylated and monofucosylated glycans positively correlated with pro-inflammatory cytokines, whereas poly and high-fucosylated glycans are negatively correlated, highlighting the important role of N-glycosylation in maintaining immune homeostasis in the female reproductive tract. An abundance of paucimannose glycans was also detected in the low mass range of CVF, which could originate from infiltrating neutrophils, monocytes and macrophages^48,55,84–86^. These glycans could be derived from a recently described noncanonical truncation N-glycosylation pathway in human neutrophils^48^. Paucimannose glycans are found on proteins in azurophilic granules of neutrophils, which can be selectively secreted upon pathogen stimulation, and their glycosylation status is involved in modulating multiple immune functions central to inflammation and infection^87–89^. The unusually high abundance of paucimannose structures found in CVF is potentially indicative of neutrophil invasion in the lower female reproductive tract, which could be used as a defence mechanism against invading pathogens. It is plausible that glycan-GBP interactions between pathogenic microbes and neutrophils leads to neutrophil degranulation, an abundance of paucimannoses, pro-inflammatory cytokine release and subsequent preterm labour. Another possible explanation for the paucimannose glycans is that they are the result of degradation by microbial glycohydrolytic enzymes^69,70^.

It should be noted that CVF is also heavily O-glycosylated and we are undertaking similar analytic approaches to characterise these glycan structures.

In summary, this study provides evidence that cervicovaginal glycans are associated with microbial composition and immune response in pregnancy where they may influence clinical outcome. Modulation of cervicovaginal glycans could thus represent a novel therapeutic strategy to prevent microbial driven preterm birth.

## Methods

### Patient recruitment and sampling

The study was conducted with approval of the NHS National Research Ethics Service (NRES) Committees London—Stanmore (REC 14/LO/0328), and in accordance with relevant guidelines, regulations, and the Declaration of Helsinki. All methods were performed in accordance with the relevant guidelines and regulations. All patients and non-pregnant women provided written informed consent. Recruitment and sampling were performed at Queen Charlotte’s and Chelsea Hospital, Imperial College Healthcare NHS Trust, London, UK. Non pregnant women were eligible if they were of reproductive age and aged 18 or over. Pregnant women at risk of preterm birth were eligible. Risk factors included having a short or open cervix, a previous preterm delivery or previous cervical treatment. Exclusion criteria included women under 18 years of age, those who had sexual intercourse within 72 h of sampling, vaginal bleeding in the preceding week, HIV or Hepatitis C positive status. Detailed maternal clinical metadata and birth outcome data was collected for all pregnant participants. CVF was sampled using the BBL™ CultureSwab™ MAXV liquid Amies swabs (Becton, Dickinson and Company, Oxford UK) for assessing microbial composition. CVF was then collected using a menstrual cup (Softdisc™, The Flex Company, USA) by placing it against the cervix for 20 min. After removal, material from both sides of the cup was retrieved by repeated pipetting of phosphate buffer saline (PBS) over each side leading to resuspension of material in a 1:5 weight: volume ratio within 30 min of collection. The suspension was distributed into separate aliquots to prevent unnecessary freeze thaw cycles. Aliquot one was reserved for glycomic profiling. Aliquot two was centrifuged (500×*g*, 10 min, 4 °C), and the supernatant was reserved for immune profiling. Samples were stored at − 80 °C until analysis.

### N-Glycan profiling of CVF samples

Methanol, acetonitrile, ammonia, chloroform, DMSO, propan-1-ol, sodium hydroxide, acetic acid were from Romil (Cambridge, UK). Idoacetic acid, sodium chloride, iodomethane, ammonium bicarbonate, EDTA, trypsin and Tris were from Merck (Poole, UK). PNGase F (cloned from Flavobacterium meningosepticum and expressed by *E. coli*), CHAPS and DTT were from Roche Applied Science (East Sussex, UK). 8 M guanidine hydrochloride (GuHCl) and Slide-A-Lyzer™ G2 Dialysis Cassettes, 3.5K MWCO were from Thermo scientific (Loughborough, UK).

Glycomic sample processing was done following the protocol detailed previously^90,91^. Briefly, CVF samples from aliquot one were sonicated in 25 mM Tris, 150 mM NaCl, 5 mM EDTA, and 1% CHAPS, pH 7.4, dialysed in dialysis cassettes, reduced by DTT, carboxymethylated by IAA, and digested by trypsin. N-glycans were released by PNGase F and were permethylated. The permethylated glycans were cleaned by C18 cartridges and freeze dried before mass spectrometry analysis. Glycan profiling was done on AB Sciex 4800 MALDI-TOF/TOF mass spectrometer. The methylated glycans were dissolved in 10 ul methanol. One 1ul of sample was mixed with 1 ul of 10 mg/ml DABP matrix in 75% ACN. The mixture was spotted on a MALDI plate for MALDI-TOF–MS analysis. The data were analysed using Data Explorer™ version 4.6 from AB Sciex, Glycoworkbench^92^ and MALDIquant^93^. The glycomic data were annotated based on monosaccharide composition derived from the molecular ion m/z value, knowledge of N-glycan biosynthetic pathways, the isotopic peak cluster patterns, the glycosylation patterns in the low and medium mass range, and MS/MS derived fragmentation. For glycans with overlapped isotopic peak clusters, the dominant one was annotated.

The statistical analysis of the glycans was done using SQLite3 and R 3.6.3 on a Linux Ubuntu 20.04.4 LTS platform. The scripts are available on GitHub (Link: https://github.com/gw110/CVF-glycosylation.git). In order to have the least interference of overlapped isotopic peaks, Glycans with 2 or 3 LacNAc units were selected for quantitative analysis to assure high accuracy of data annotation and quantitation. The relative intensities of different glycan subgroups were calculated using SQLite3. The results were exported and visualized using ggplot2 in R 3.6.3. The correlation of glycan relative intensities with cytokines was done using the linear regression model. Pearson product-moment correlation coefficient and p value were calculated using the cor.test function in R. Box plot and two tailed Student’s t test was used to compare glycosylation between sample groups.

Glycans were further analysed following a glycotope-centric glycomic analysis based on a nanoLC-MS^2^-product dependent-MS^3^ data acquisition workflow, previously established using the advanced Orbitrap Fusion Tribrid MS platform^94^. In essence, this takes advantage of the high acquisition speed, high mass accuracy and high sensitivity of the MS instrument to perform a comprehensive MS^2^ mapping of the terminal glycotope based on preferred cleavage at the HexNAc of permethylated glycans. The respective intensity of each characteristic oxonium ion measured at 5 ppm mass accuracy or less can be summed from all glycan MS^2^ spectral acquired throughout the LC–MS/MS run to provide a relative abundance index for the glycotope it represents. In cases when isomeric glycotopes need to be differentiated, as for the Lewis and H epitopes in this work, the MS^2^ ion can be coupled with a further MS^3^ event. The diagnostic MS^3^ ions resulting from specifically eliminating the C3-substituent of the HexNAc^+^ is sufficient to resolve the isomeric difference and their relative intensity can in turn provide a semi-quantitative estimation of the relative abundance of each isomeric constituent of that particular glycotope.

### Bacterial DNA extraction and metataxonomic profiling

Extraction of DNA from CVF samples and sequencing of 16S rRNA hyper variable regions was performed as previously described^46^. Briefly, V1-V2 hyper variable regions of bacterial 16S rRNA genes were amplified using a mixed forward primer set (28f.-YM) consisting of the following primers mixed at a 4:1:1:1 ratio; 28F-Borrellia GAGTTTGATCCTGGCTTAG; 28F-Chlorflex GAATTTGATCTTGGTTCAG; 28F-Bifido GGGTTCGATTCTGGCTCAG; 28F GAGTTTGATCNTGGCTCAG. The reverse primer consisted of; 388R TGCTGCCTCCCGTAGGAGT^95^. Amplified products were then pooled equimolar and each pool was size selected in two rounds using Agencourt AMPure XP (BeckmanCoulter, Indianapolis, Indiana) in a 0.7 ratio for both rounds. These were then quantified using the Quibit 2.0 Fluorometer (Life Technologies) and loaded on an Illumina MiSeq (Illumina, Inc. San Diego, California) 2 × 300 flow cell at 10 pM. All sequencing was performed at Research and Testing Laboratory (Lubbock, TX, USA).

Trimming of primer sequences was performed using Cutadapt^96^ and QC performed using FastQC^97^. Resulting amplicon sequence variant (ASV) counts were calculated for each sample using the Qiime2 pipeline^98^. DADA2 was used for denoising^99^ and taxonomic classification of sequences to species level was performed using the STIRRUPS reference database^100^. Samples were classified into vaginal community state types (CSTs) using the VAginaL community state typE Nearest CentroId classifier (VALENCIA)^47^. Using this standardised approach, CST 1 communities are characterised by *Lactobacillus crispatus* dominance and can be further subdivided into subtypes CST 1-A (almost complete dominance by *L. crispatus*) and CST 1-B (less *L. crispatus*, but still the majority). CST IV communities have a low relative abundance of Lactobacillus spp. and include subtypes CST IV-A, CST IV-B, and CST IV-C, depending on the relative abundances of *G. vaginalis*, *A. vaginae*, and BVAB1, respectively.

### Immune profiling of CVF samples

The stored supernatant from the CVF (aliquot two) was thawed on ice and used for Luminex® immunoassays to quantify cytokines by multiplexed bead-based immunoassays. The concentrations of 10 cytokines (IL-1β, IL-18, IL-6, IL-2, IL-4, IL-5, IL-10, IFN-γ, GM-CSF and TNF-α) were measured on a multiplex plate Human Premixed Multi-Analyte Kit (R&D Systems), following manufacturer’s instructions. IL-8 was measured on a single-plex Human Premixed Analyte Kit (R&D Systems), following a 10-fold dilution using Calibrator Diluent RD6-52. All standards and samples were run in duplicate. Concentrations of IL-2, IL-5, IFN-γ, and GM-CSF were undetectable in all samples. IL-4, IL-10 and TNF-α were only detectable in a proportion of samples.

## Supplementary Information

Supplementary Information.

## Data Availability

The R and SQLite3 scripts for glycan quantitative data analysis are available on GitHub (Link: https://github.com/gw110/CVF-glycosylation.git).
