# Supplementary material for: N-glycosylation of cervicovaginal fluid reflects microbial community, immune activity, and pregnancy status
Source: Sci Rep. 2022 Oct 10;12:16948. doi: 10.1038/s41598-022-20608-7 (PMC9551102; doi:10.1038/s41598-022-20608-7)
Supplement: Supplementary file 1 — Supplementary Information. [file 41598_2022_20608_MOESM1_ESM.docx]

**Supplementary Data**

Supplementary Table S1. Details of CVF samples collected for glycomic analysis

| Sample code | Sample collection (Weeks + Days) | Gestation (Weeks + Days) | Outcome | Ethnicity | Blood group | CST | IL1 beta  (pg/ml) | IL 6  (pg/ml) | IL 18  (pg/ml) | IL 8  (pg/ml) |
| --- | --- | --- | --- | --- | --- | --- | --- | --- | --- | --- |
| NP1 | 0+0 | 0+0 | Non-pregnant | Caucasian | O | Not Available | 9.24 | 3.78 | 0.79 | 1108.13 |
| NP2 | 0+0 | 0+0 | Non-pregnant | South Asian | A | Not Available | 2.76 | 1.25 | 0.68 | 862.41 |
| NP3 | 0+0 | 0+0 | Non-pregnant | African | A | Not Available | 156.27 | 19.6 | 2.96 | 2938.6 |
| NP4 | 0+0 | 0+0 | Non-pregnant | Caucasian | A | Not Available | 15.94 | 2.47 | 0.84 | 285.47 |
| P1 | 15+3 | 39+0 | Term | Caucasian | O | I-B | 2012.4 | n.d | 1.01 | 7601.02 |
| P2 | 22+5 | 39+3 | Term | African | B | I-A | 29.3 | 8.39 | 0.9 | 636.22 |
| P3 | 20+4 | 39+3 | Term | African | O | IV-B | 91.1 | 1.35 | 1.5 | 1048.06 |
| P4 | 20+5 | 27+3 | Preterm | South Asian | B | I-A | 1200.78 | 285.65 | 2.23 | 5727.55 |
| P5 | 23+5 | 31+3 | Preterm | African | B | IV-B | 20705.32 | 7.53 | 64.2 | 1703.05 |
| P6 | 21+3 | 22+4 | Preterm | South Asian | B | IV-B | 3799.6 | 1374.41 | 43.88 | 9651.97 |

n.d: not detected


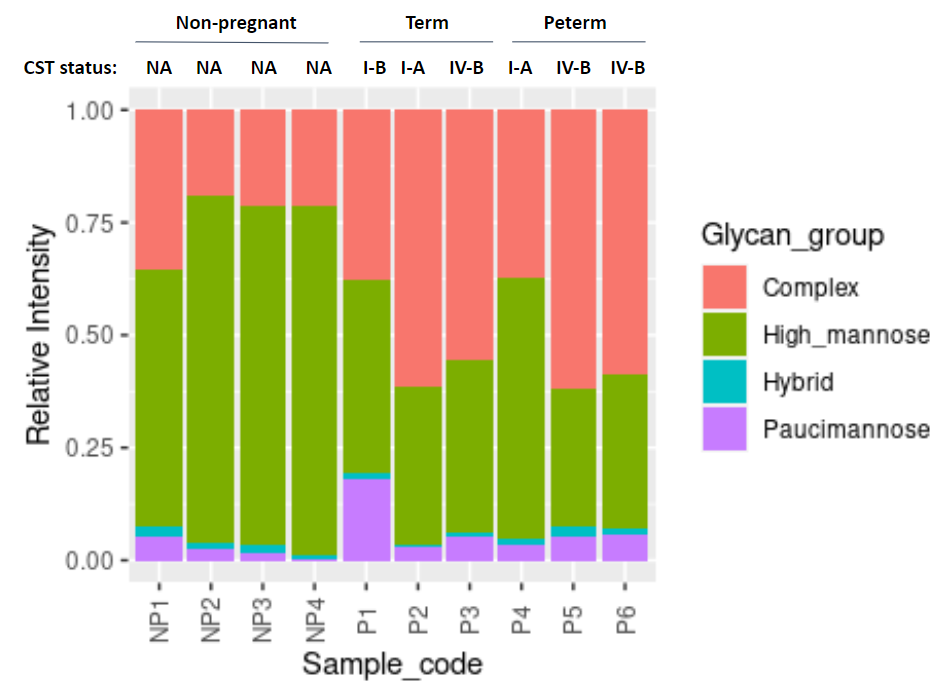


Supplementary Fig. S1. Relative abundances of paucimannose, high mannose, hybrid and complex glycans in different CVF samples.


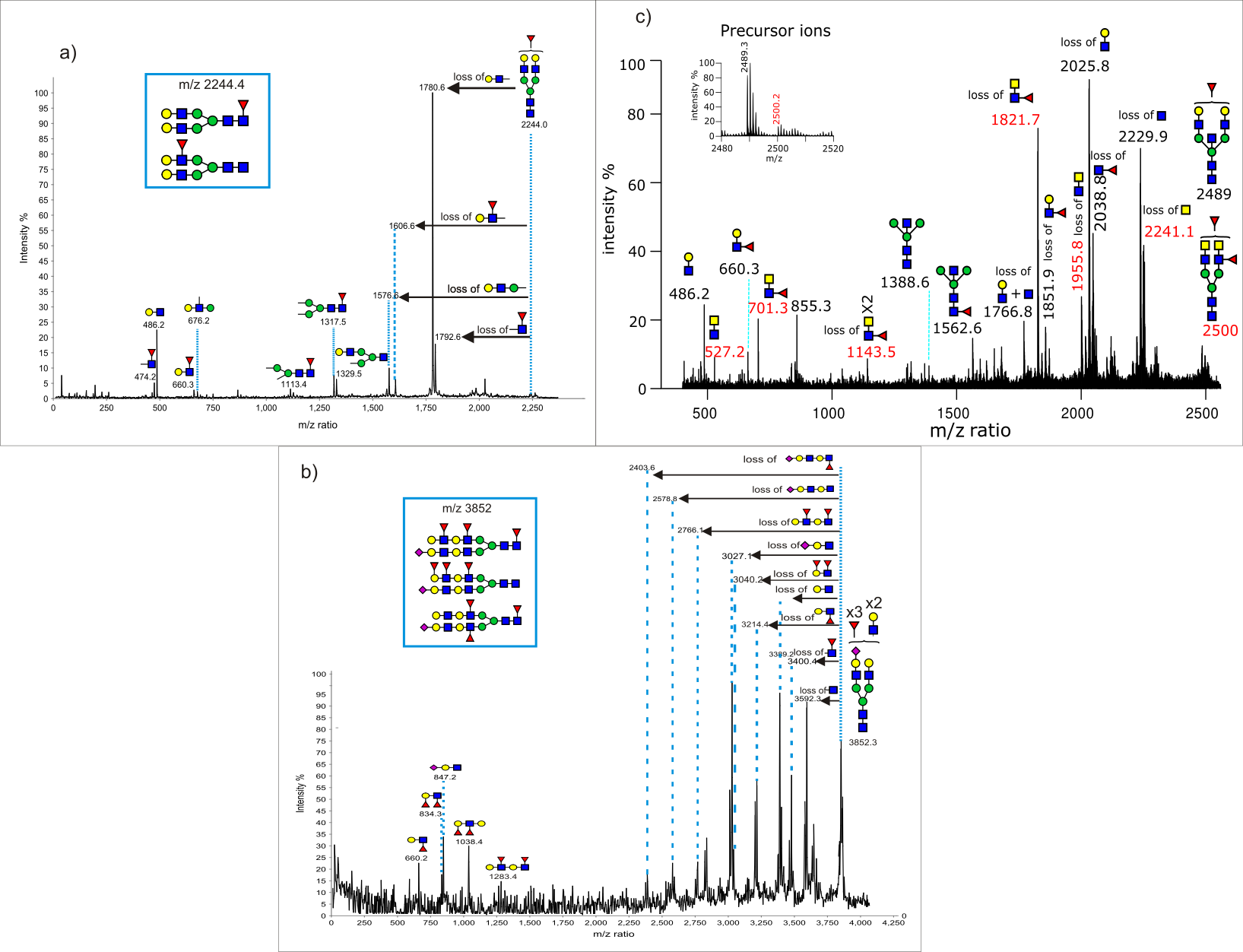


Supplementary Fig. S2.MS/MS analysis of CVF N-glycans. a) MS/MS of the glycan at m/z 2040 confirms the presence of mixed core fucosylation and terminal fucosylation. b) MS/MS of the glycan at m/z 3852 illustrated the presence of extended antennae on glycans with poly LacNAcs. c) MS/MS of the glycan at m/z 2500 showed the presence of LacdiNAc structures (m/z values in red). A glycan with a much higher intensity nearby (m/z 2489) was co-selected for MS/MS fragmentation, and the m/z values from this glycan are in black.

1. **m/z 1000-2000**

**
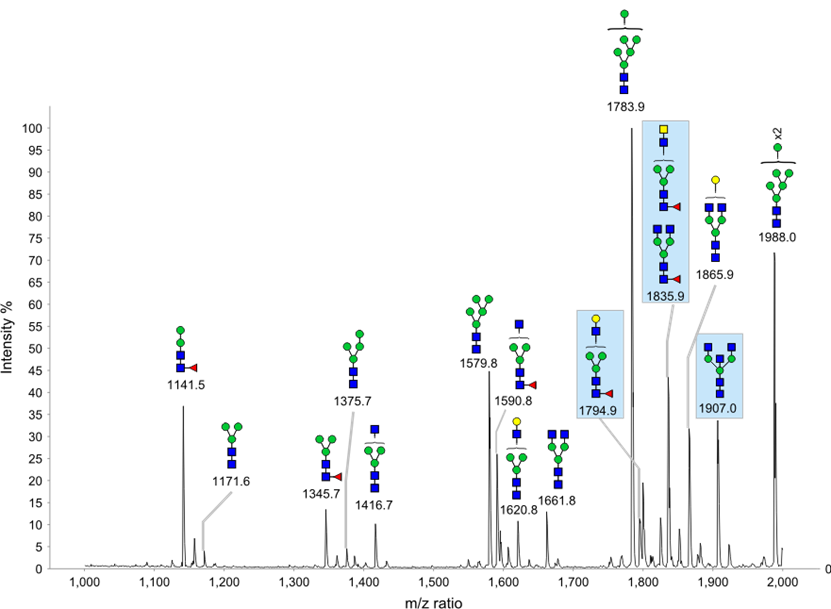
**

1. **m/z 2000-2500**


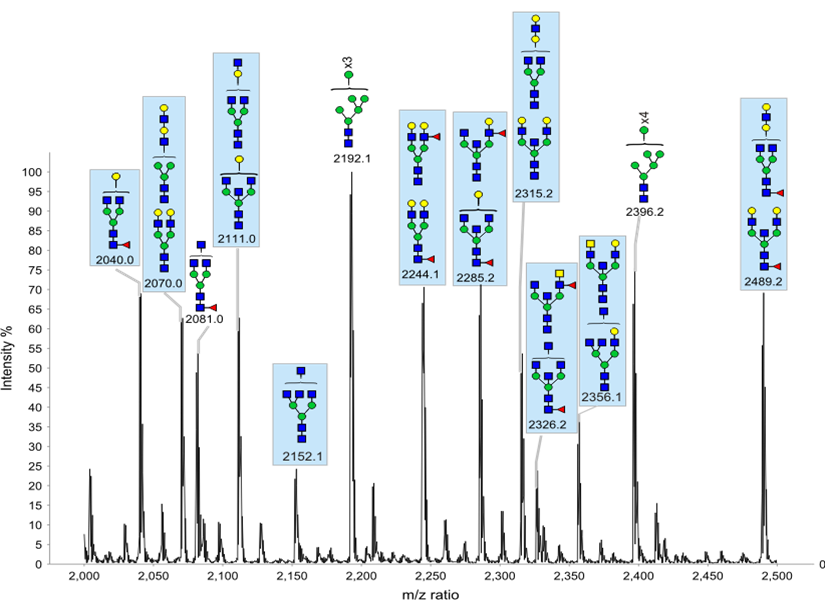


1. **m/z 2500-3000**


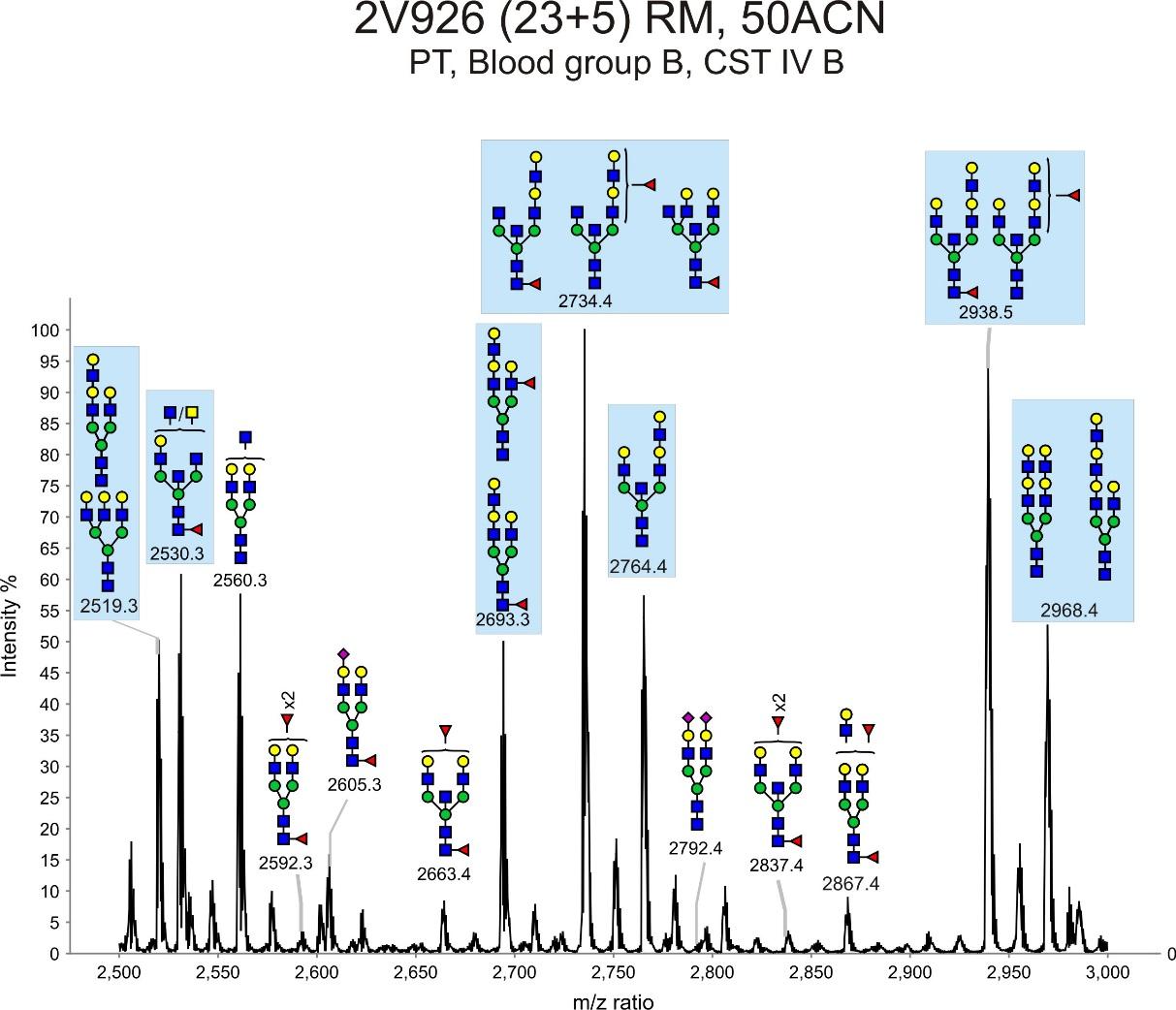
 **d) m/z 3000-3500**


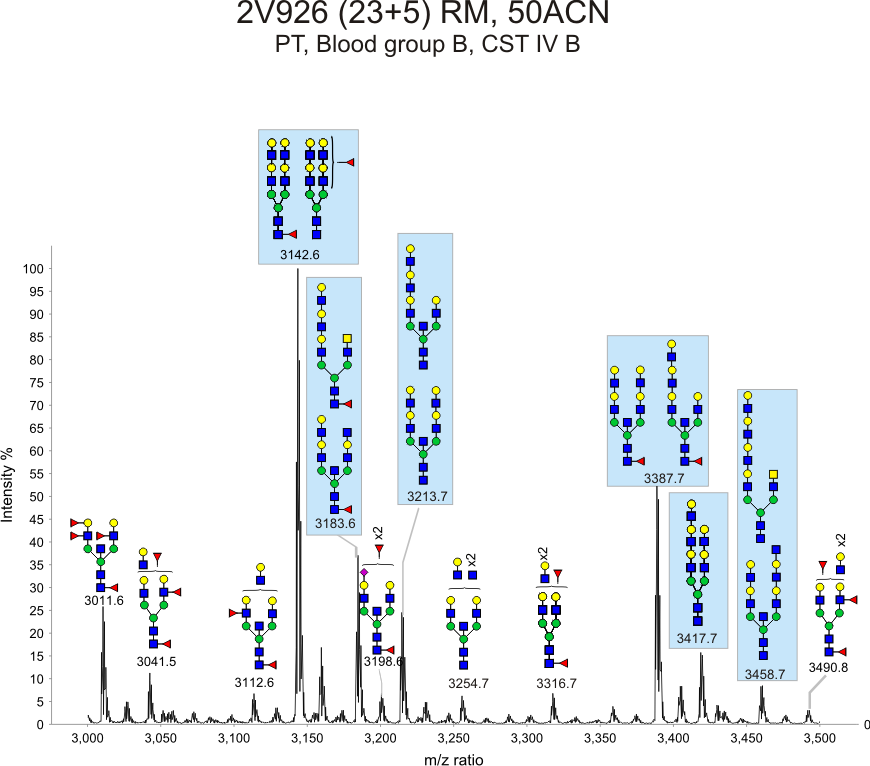


**e) m/z 3500-4000**


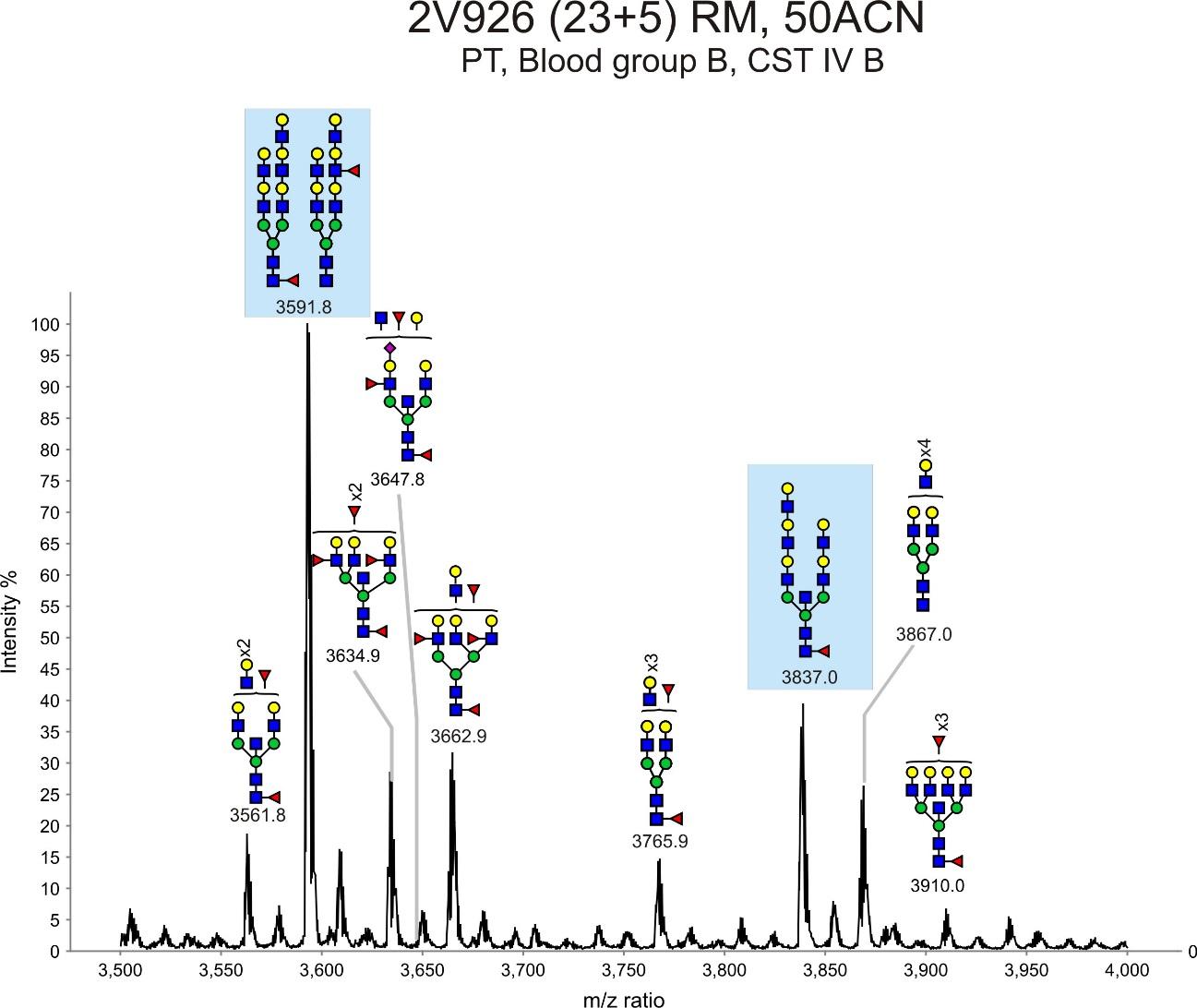


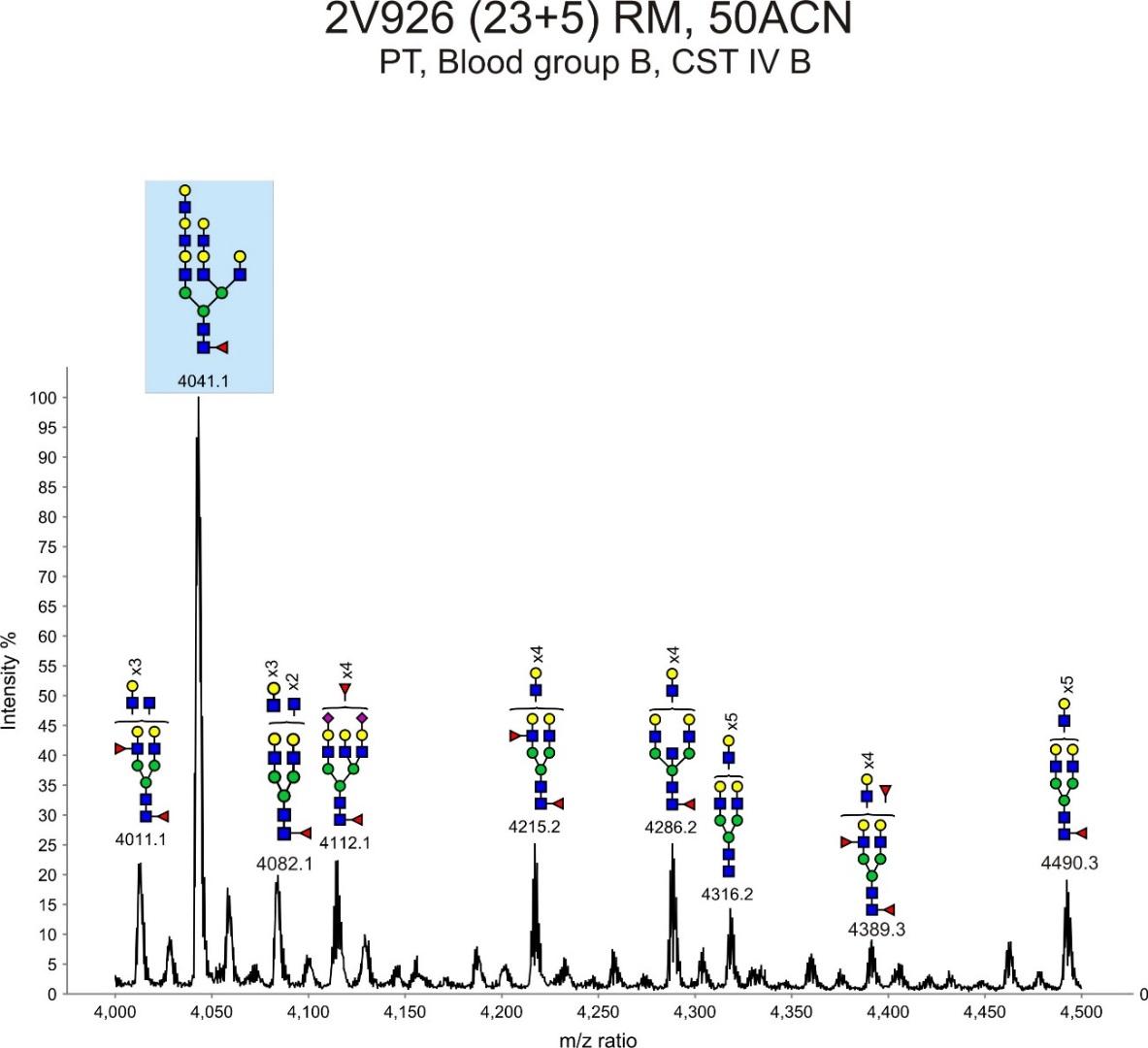
**f) m/z 4000-4500**


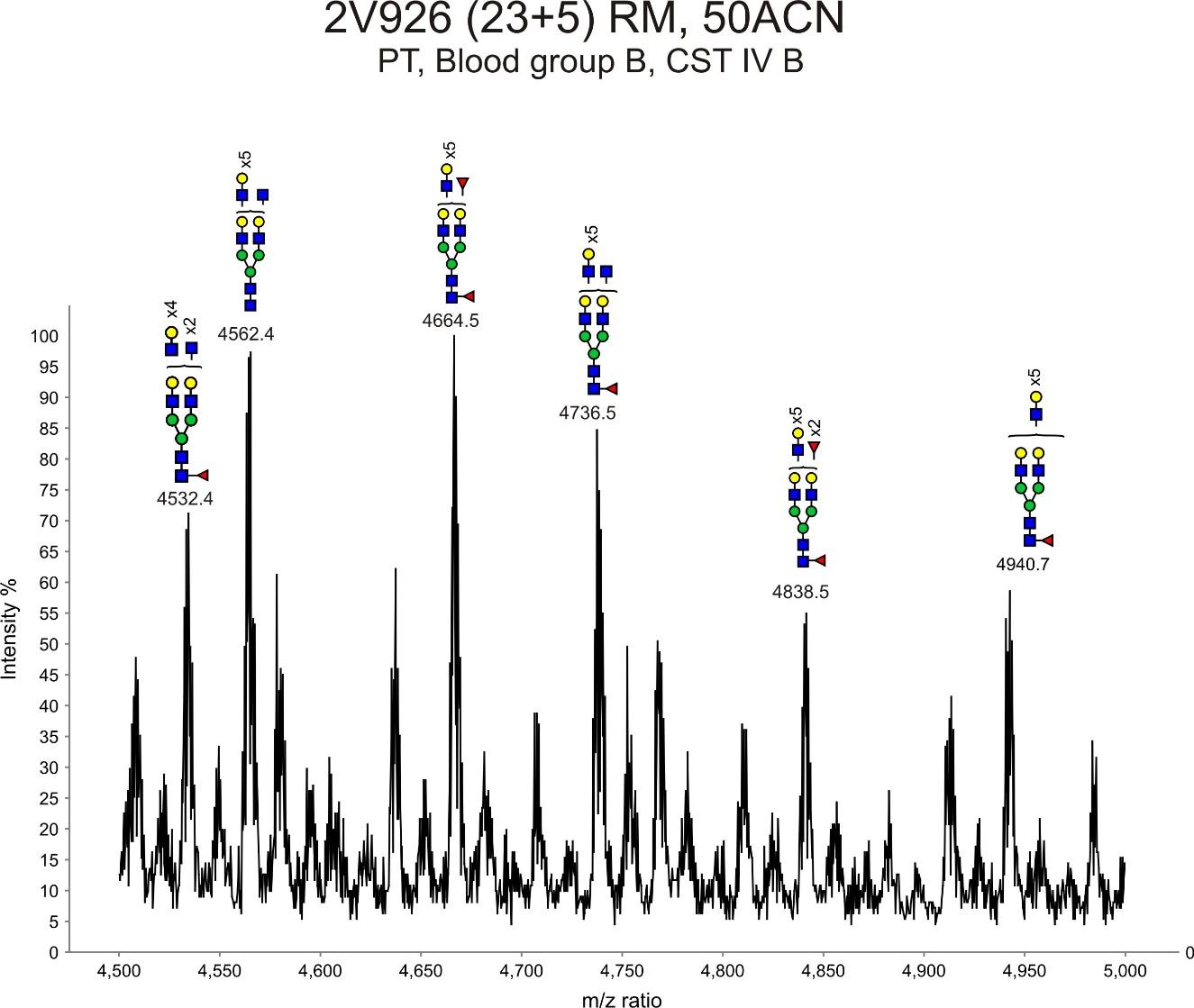
**g) 4500-5000**

Supplementary Fig. S3. MALDI-TOF mass spectra of N-glycans from CVF donor with blood group B and CST IV B, who delivered preterm at 31 weeks and 3 days (sample P5). Regions from the MALDI-MS spectra are expanded for clarity. Panel **a)**, *m/z* 1000-2000 Panel **b)**, *m/z* 2000-2500; Panel **c)**, *m/z* 2500-3000; Panel **d)**, *m/z* 3000-3500; Panel **e)**, *m/z* 3500-4000; Panel **f)**, *m/z* 4000-4500, **g)**, *m/z* 4500-5000. The CVF N-glycans were released by PNGase F, permethylated, and subsequently subjected to Sep-Pak clean up ("Materials and Methods"). Data were acquired in the positive ion mode to give [M+Na]^+^ molecular ions. Peak assignments are based on 12C isotopic composition together with knowledge of the biosynthetic pathways, and structures in light blue boxes were confirmed by MS/MS. Residues above a bracket have not had their location unequivocally defined.

1. **m/z 1000-2000**


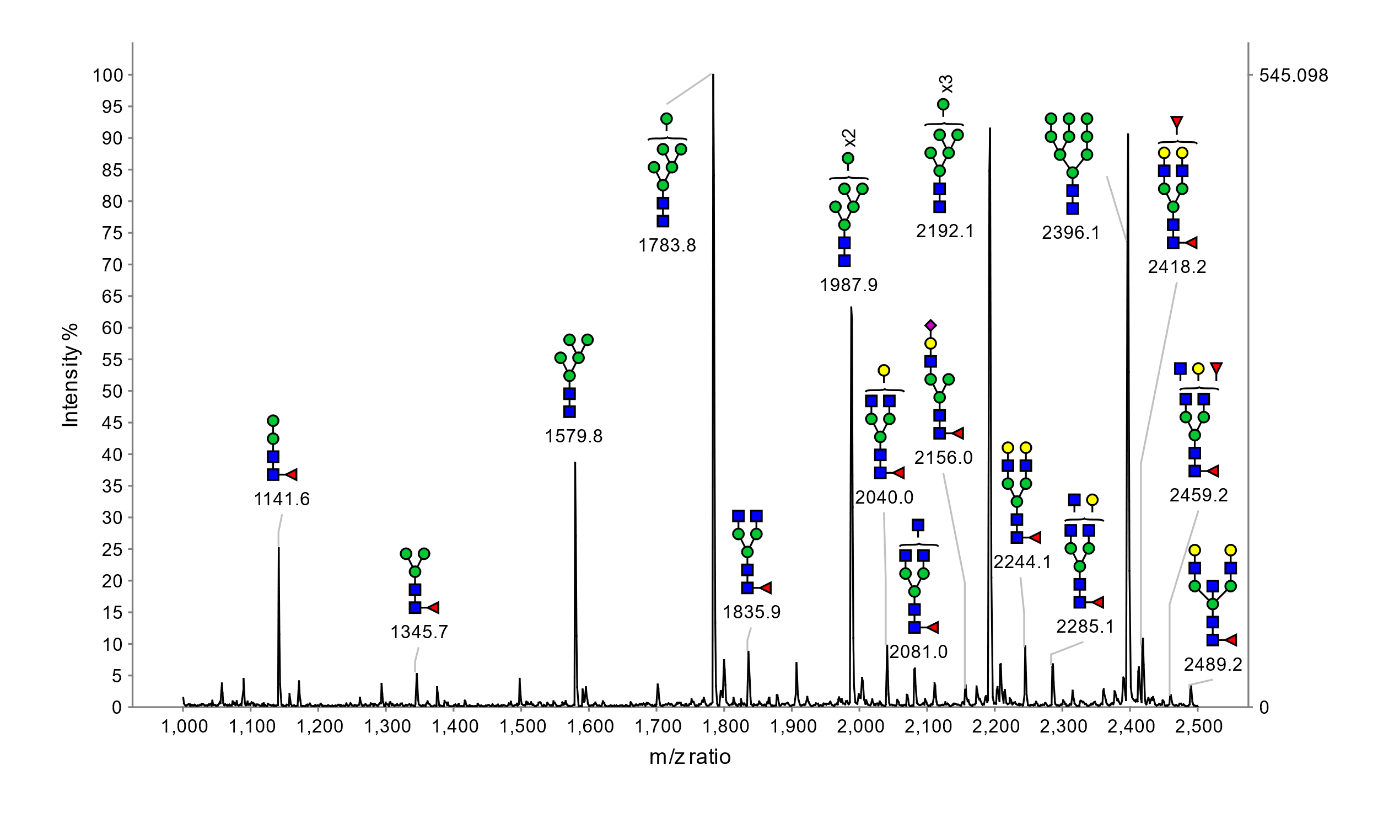


1. **m/z 2000-3000**


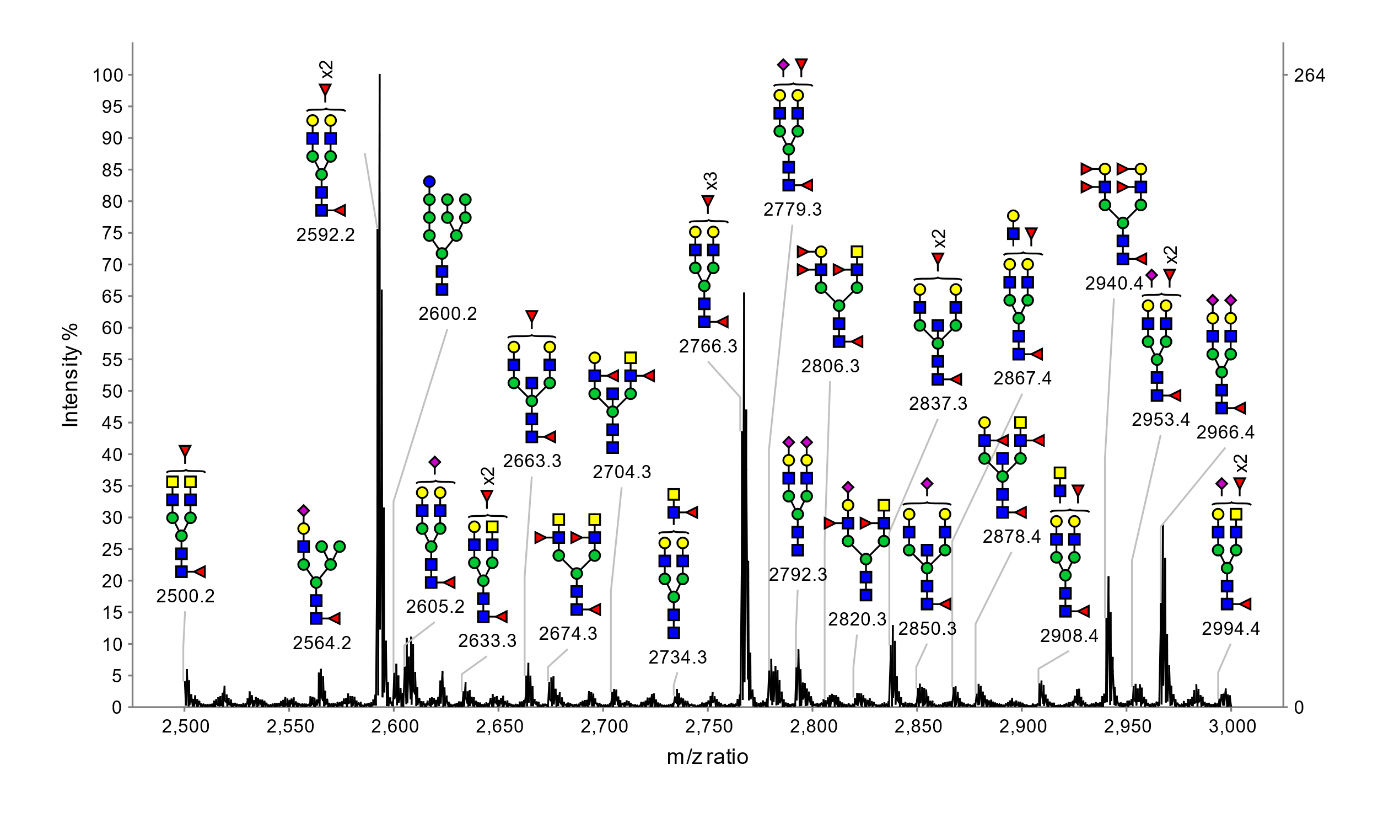


1. **m/z 3000-3500**


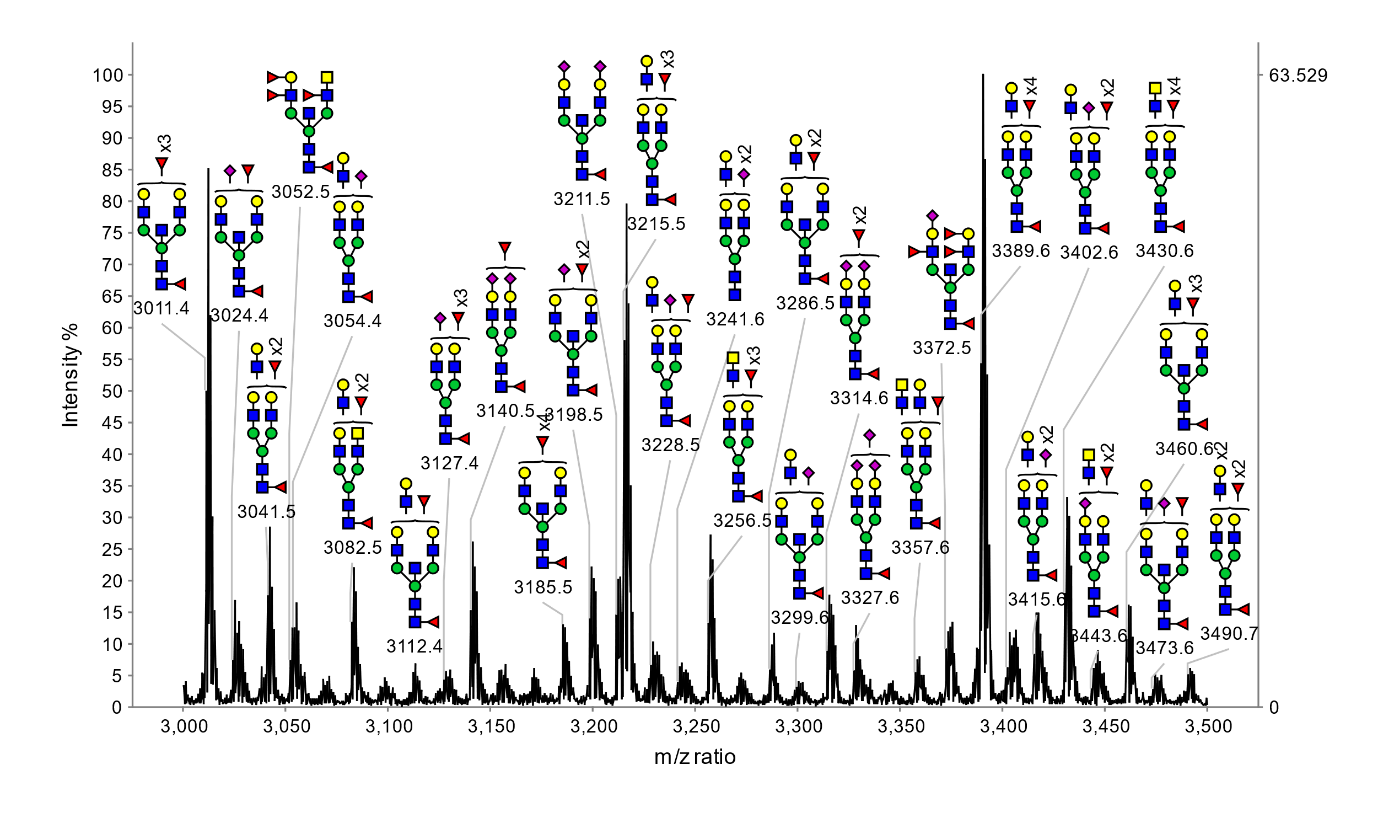


1. **m/z 3500-4000**


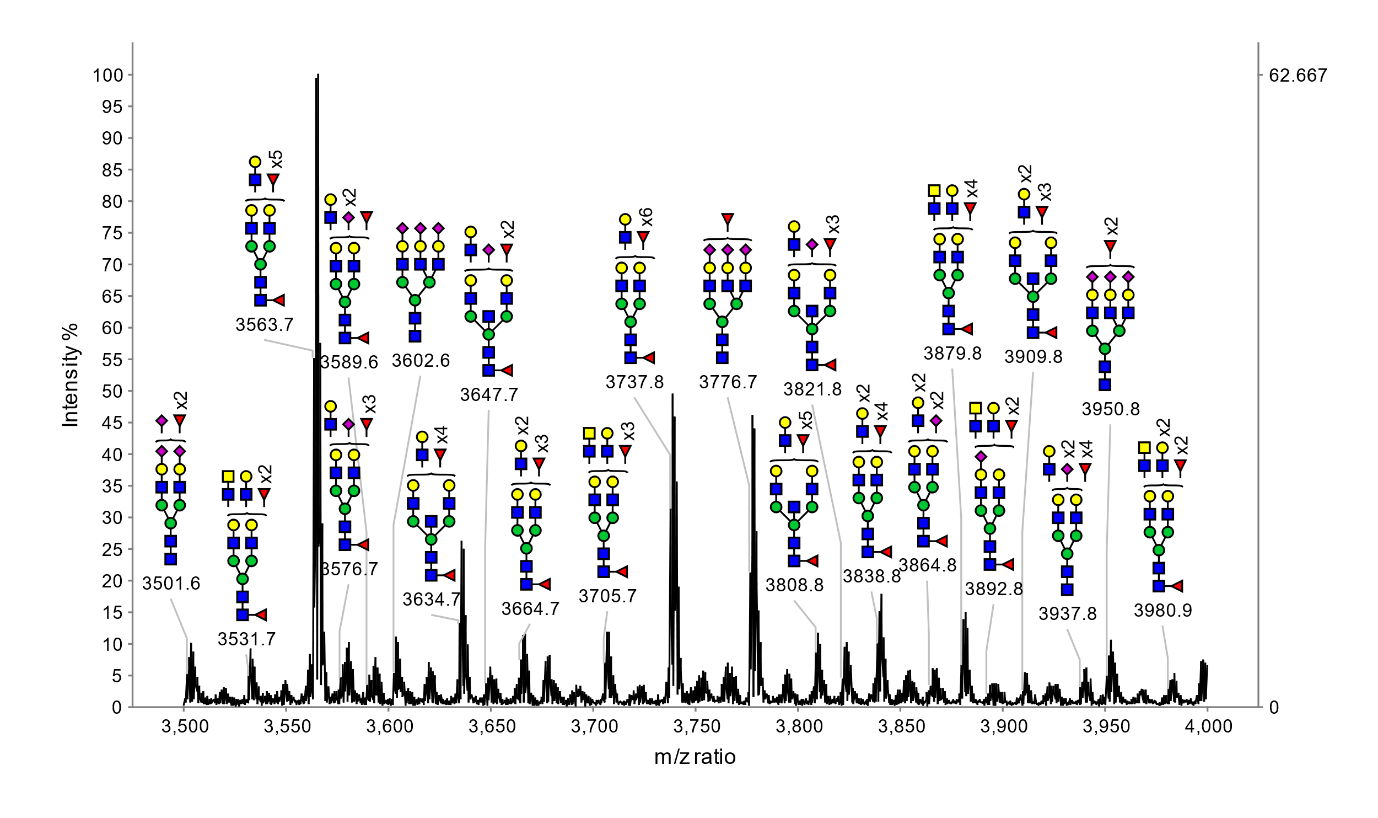


1. **m/z 4000-4500**


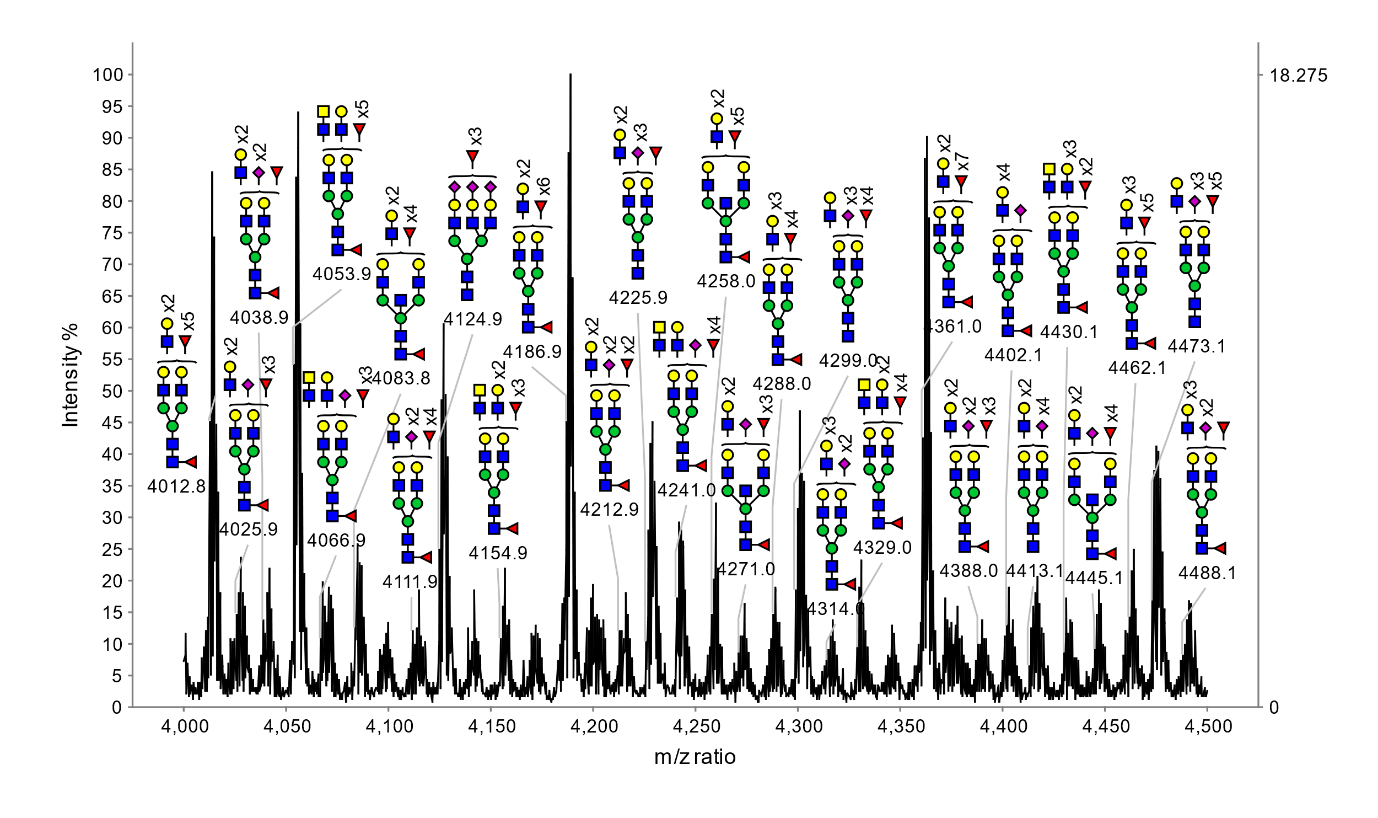


1. **m/z 4500-5000**


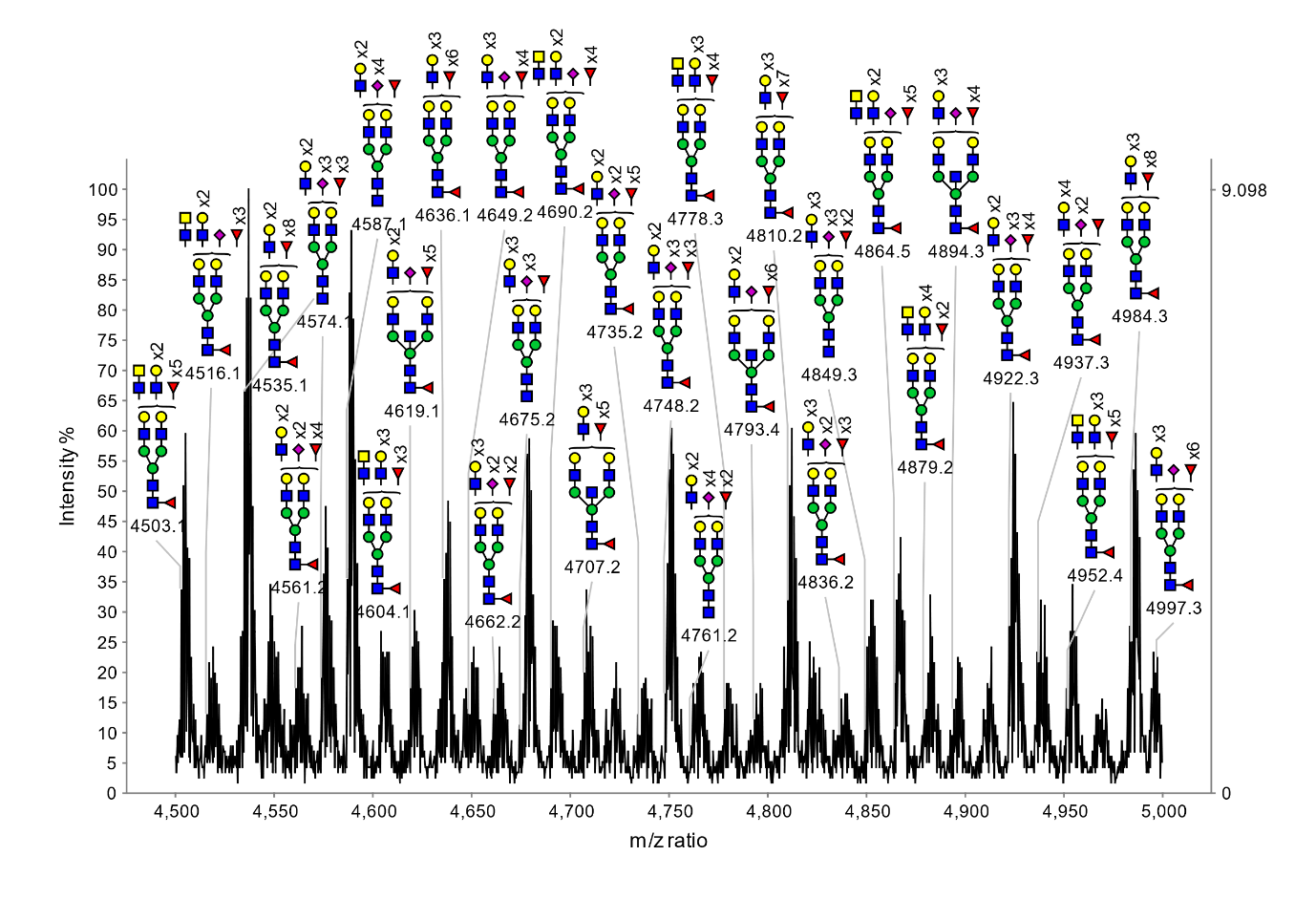


Supplementary Fig. S4. N-glycans MALDI-TOF mass spectra of CVF of a non-pregnant donor (sample NP1). Regions from the MALDI-MS spectra are expanded for clarity. Panel **a)**, *m/z* 1000-2000 Panel **b)**, *m/z* 2000-3000; Panel **c)**, *m/z* 3000-3500; Panel **d)**, *m/z* 3500-4000; Panel **e)**, *m/z* 4000-4500; Panel **f)**, *m/z* 4500-5000. The CVF N-glycans were released by PNGase F, permethylated, and subsequently subjected to Sep-Pak clean up ("Materials and Methods"). Data were acquired in the positive ion mode to give [M+Na]^+^ molecular ions. Peak assignments are based on 12C isotopic composition together with knowledge of the biosynthetic pathways. Residues above a bracket have not had their location unequivocally defined.


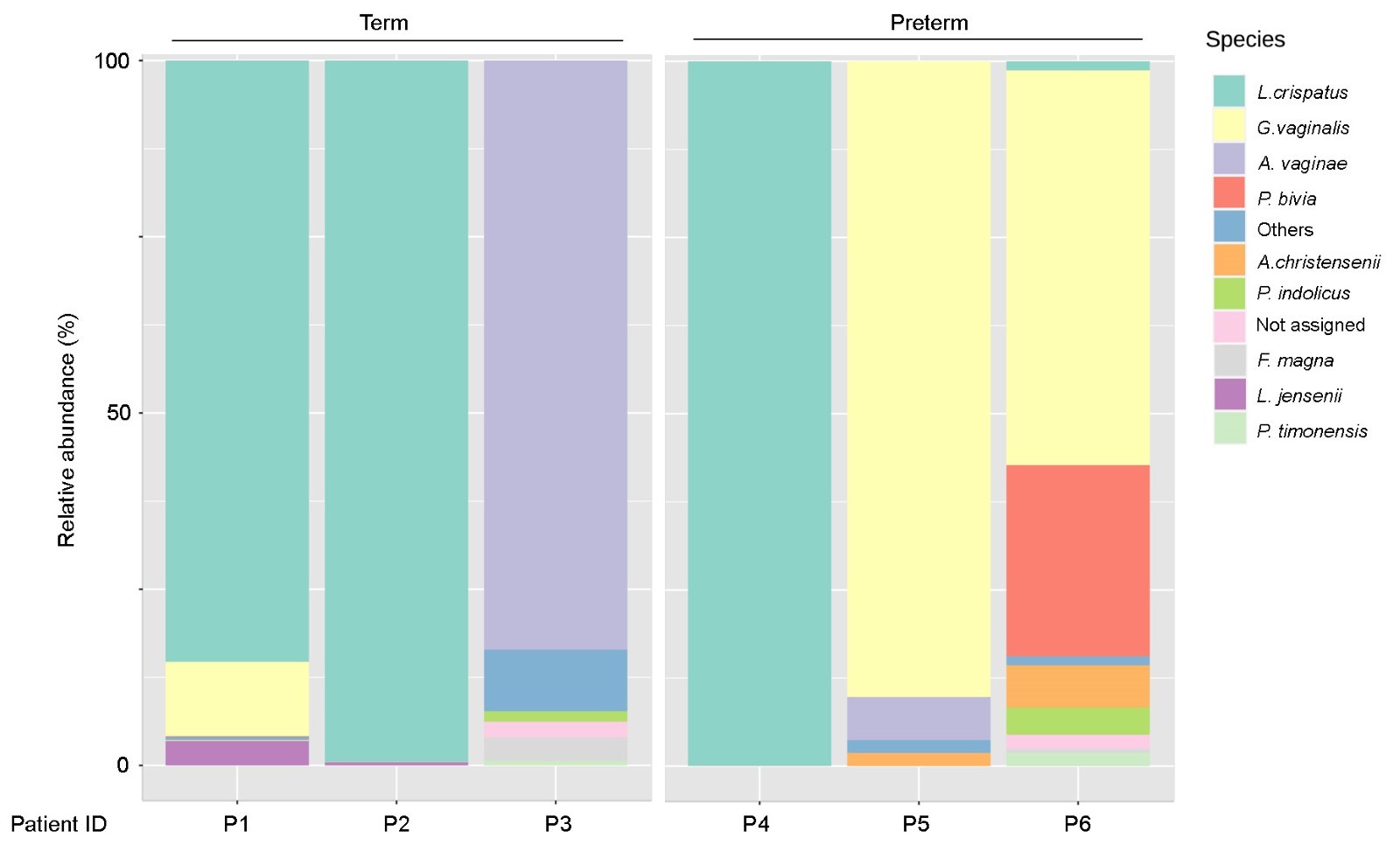


Supplementary Fig. S5. Stacked bar chart described relative abundance of major species in each of the pregnant donors. The average sequence read counts generated per sample was 15275 (minimum 6199, maximum 26203). Species are presented in order of the higher overall relative abundance within the dataset (*L. crispatus*) to the lowest and are stratified on the basis of pregnancy outcome (term or preterm).


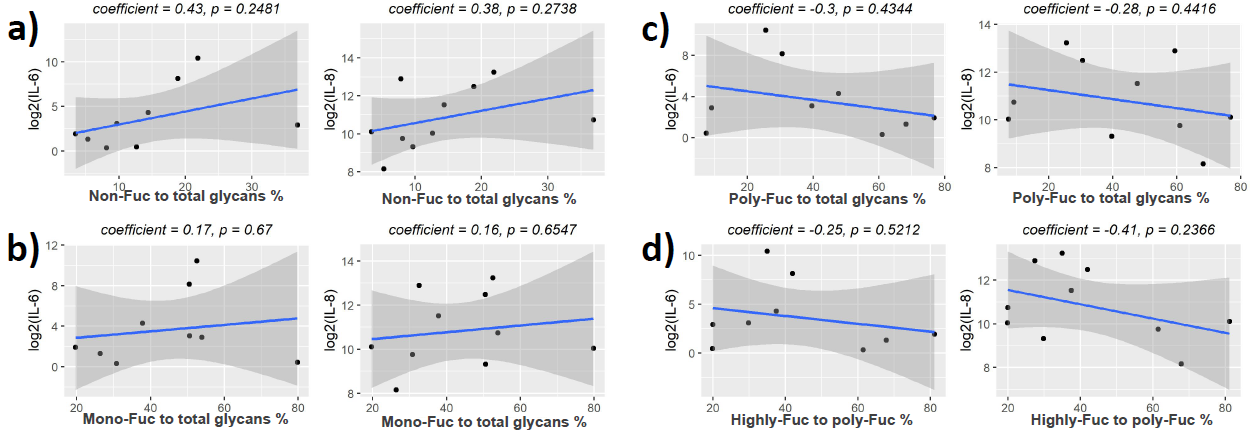


Supplementary Fig. S6. Correlation of fucosylation to IL-6 beta and IL-8 for glycans with 2 LacNAc units. Pearson's product-moment correlation was used for statistical analysis. a) relative intensity of non-fucosylated glycans to the intensity of all glycans. b) relative intensity of mono-fucosylated glycans to the intensity of all glycans. c) relative intensity of poly-fucosylated glycans to the intensity of all glycans. d) relative intensity of highly fucosylated glycans to the intensity of poly-fucosylated glycans. Highly fucosylated glycans were defined as those with at least 3 Fuc per glycan. Poly-fucosylated glycans were defined as those with at least 2 Fuc per glycan.


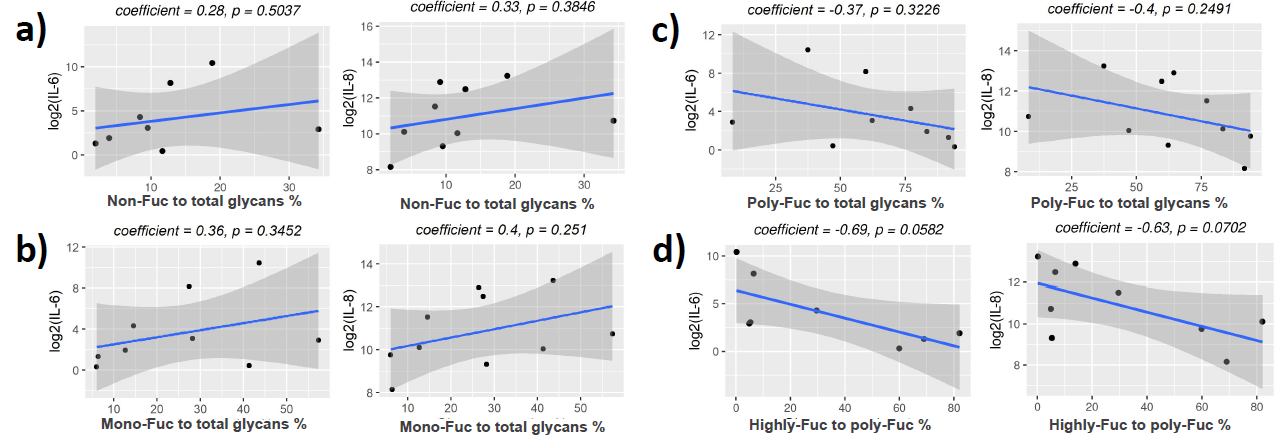


Supplementary Fig. S7. Correlation of fucosylation to IL-6 beta and IL-8 for glycans with 3 LacNAc units. Pearson's product-moment correlation was used for statistical analysis. a) relative intensity of non-fucosylated glycans to the intensity of all glycans. b) relative intensity of mono-fucosylated glycans to the intensity of all glycans. c) relative intensity of poly-fucosylated glycans to the intensity of all glycans. d) relative intensity of highly fucosylated glycans to the intensity of poly-fucosylated glycans. Highly fucosylated glycans were defined as those with at least 4 Fuc per glycan. Poly-fucosylated glycans were defined as those with at least 2 Fuc per glycan.


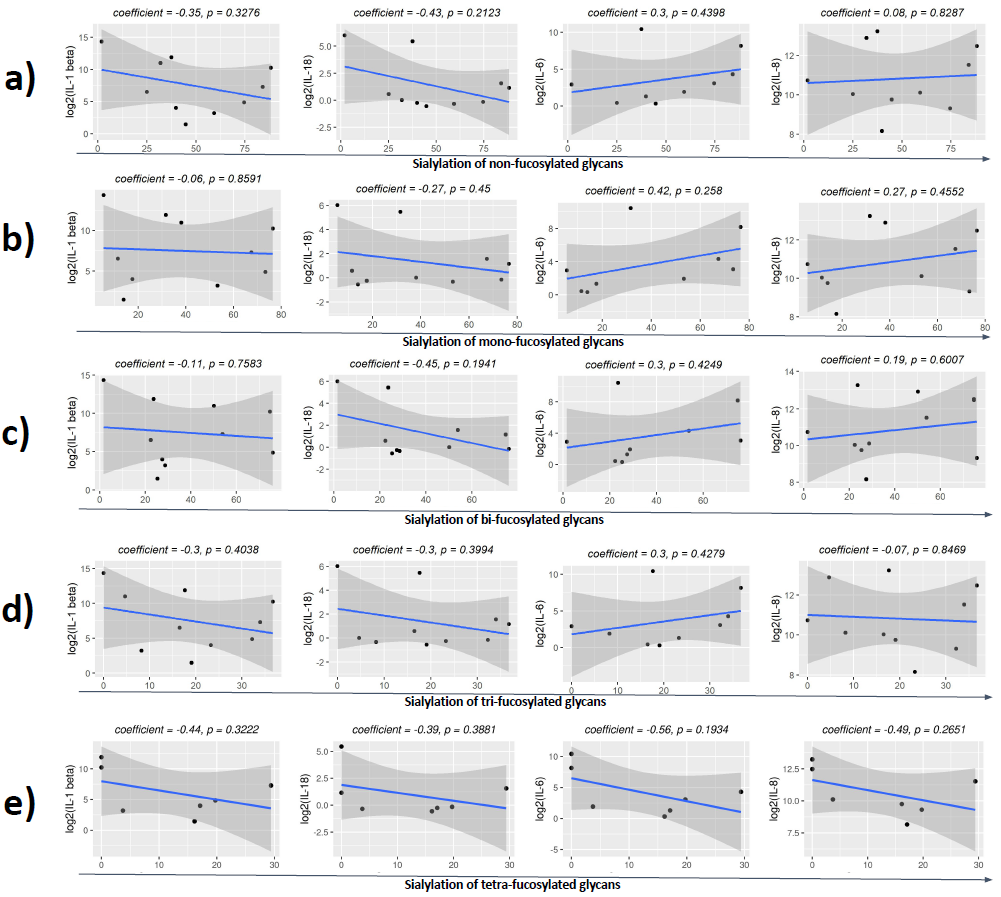


Supplementary Fig. S8. Correlation of sialylation to pro-inflammatory cytokines for glycans with 2 LacNAc units. Pearson's product-moment correlation was used for statistical analysis. For each glycan group, the proportion of sialylated glycans to all glycan intensity was calculated as the glycan proportion %. **a)** sialylation of non-fucosylated glycans. **b)** sialylation of mono-fucosylated glycans. **c)** sialylation of bi-fucosylated glycans. **d)** sialylation of tri-fucosylated glycans. **e)** sialylation of tetra-fucosylated glycans.


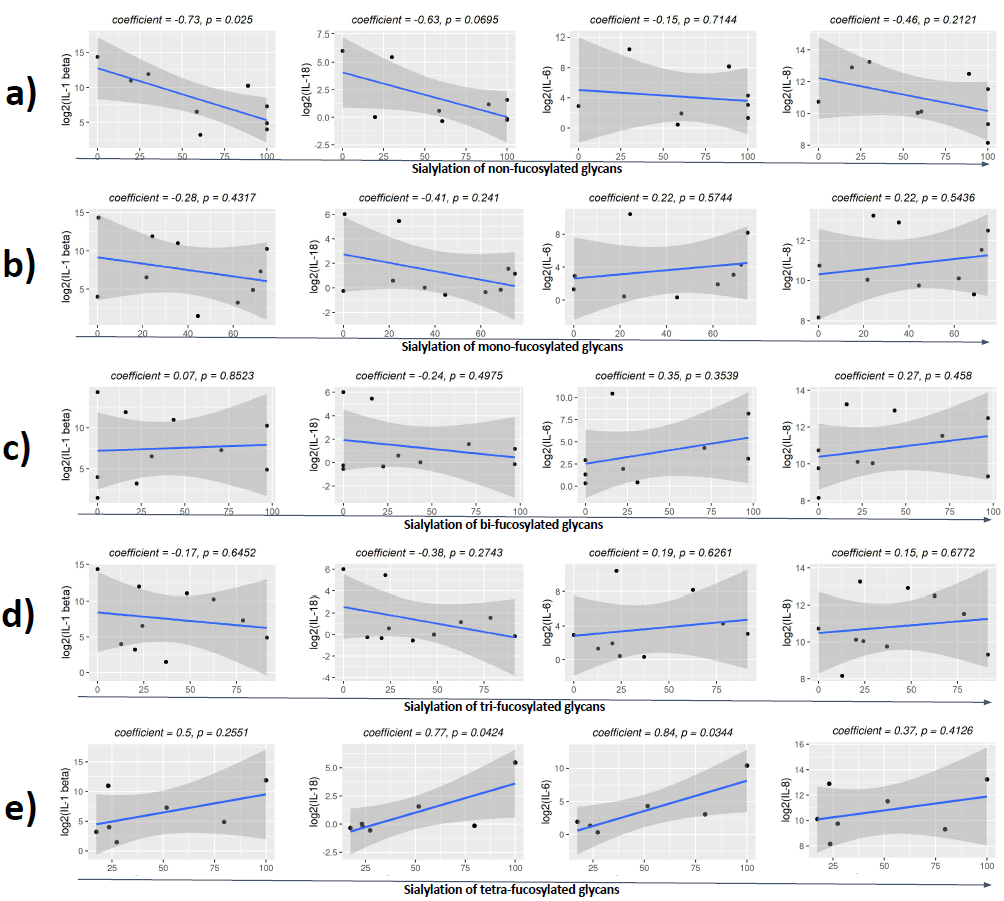


Supplementary Fig. S9. Correlation of sialylation to pro-inflammatory cytokines for glycans with 3 LacNAc units. Pearson's product-moment correlation was used for statistical analysis. For each glycan group, the proportion of sialylated glycans to all glycan intensity was calculated as the glycan proportion %. **a)** sialylation of non-fucosylated glycans. **b)** sialylation of mono-fucosylated glycans. **c)** sialylation of bi-fucosylated glycans. **d)** sialylation of tri-fucosylated glycans. **e)** sialylation of tetra-fucosylated glycans.


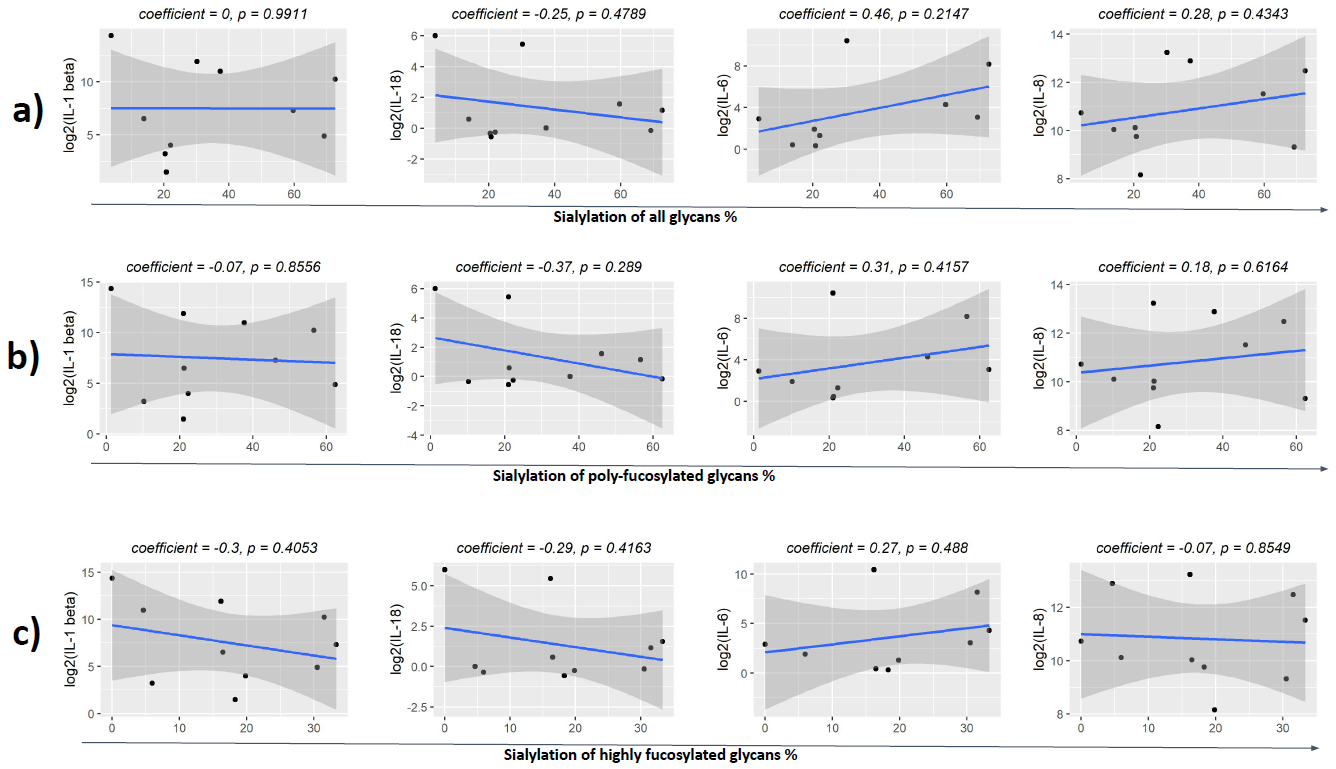


Supplementary Fig. S10. Correlation of sialylation to pro-inflammatory cytokines for glycans with 2 LacNAc units. Pearson's product-moment correlation was used for statistical analysis. For each glycan group, the proportion of sialylated glycans to all glycan intensity was calculated as the glycan proportion %. **a)** sialylation of all glycans. **b)** sialylation of poly-fucosylated glycans. **c)** sialylation of highly-fucosylated glycans. Highly fucosylated glycans were defined as those with at least 3 Fuc per glycan. Poly-fucosylated glycans were defined as those with at least 2 Fuc per glycan.


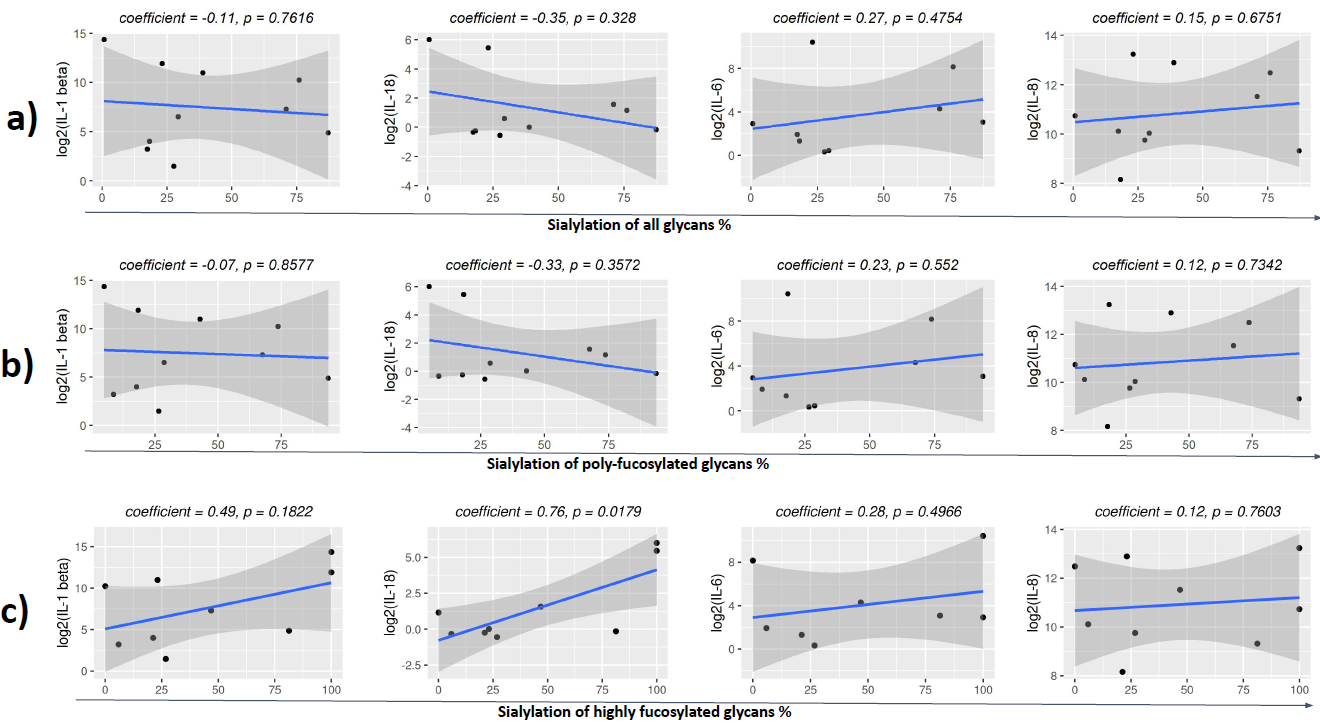


Supplementary Fig. S11. Correlation of sialylation to pro-inflammatory cytokines for glycans with 3 LacNAc units. Pearson's product-moment correlation was used for statistical analysis. For each glycan group, the proportion of sialylated glycans to all glycan intensity was calculated as the glycan proportion %. **a)** sialylation of all glycans. **b)** sialylation of poly-fucosylated glycans. **c)** sialylation of highly-fucosylated glycans. Highly fucosylated glycans were defined as those with at least 4 Fuc per glycan. Poly-fucosylated glycans were defined as those with at least 2 Fuc per glycan.

| **m/z** | **Glycan composition** |
| --- | --- |
| 5012.523 | Hex_10_HexNAc_9_dHex_4_ |
| 5025.518 | Hex_10_HexNAc_9_NeuAc_1_dHex_2_ |
|  | Hex_7_HexNAc_8_dHex_9_ |
| 5038.513 | Hex_10_HexNAc_9_NeuAc_2_ |
| 5068.549 | Hex_8_HexNAc_8_NeuAc_1_dHex_6_ |
| 5083.56 | Hex_10_HexNAc_10_dHex_3_ |
| 5096.544 | Hex_7_HexNAc_6_NeuAc_3_dHex_6_ |
| 5096.555 | Hex_10_HexNAc_10_NeuAc_1_dHex_1_ |
| 5098.559 | Hex_9_HexNAc_8_NeuAc_1_dHex_5_ |
| 5109.539 | Hex_7_HexNAc_6_NeuAc_4_dHex_4_ |
| 5113.57 | Hex_11_HexNAc_10_dHex_2_ |
| 5124.55 | Hex_9_HexNAc_8_NeuAc_3_dHex_1_ |
| 5126.565 | Hex_11_HexNAc_10_NeuAc_1_ |
|  | Hex_8_HexNAc_9_dHex_7_ |
| 5141.565 | Hex_10_HexNAc_8_NeuAc_2_dHex_2_ |
| 5154.597 | Hex_10_HexNAc_11_dHex_2_ |
| 5167.581 | Hex_7_HexNAc_7_NeuAc_3_dHex_5_ |
| 5186.612 | Hex_10_HexNAc_9_dHex_5_ |
| 5197.591 | Hex_8_HexNAc_7_NeuAc_3_dHex_4_ |
| 5199.607 | Hex_10_HexNAc_9_NeuAc_1_dHex_3_ |
| 5212.602 | Hex_10_HexNAc_9_NeuAc_2_dHex_1_ |
| 5227.638 | Hex_9_HexNAc_10_dHex_5_ |
| 5242.638 | Hex_8_HexNAc_8_NeuAc_1_dHex_7_ |
| 5259.653 | Hex_9_HexNAc_8_dHex_8_ |
| 5270.633 | Hex_7_HexNAc_6_NeuAc_3_dHex_7_ |
| 5270.644 | Hex_10_HexNAc_10_NeuAc_1_dHex_2_ |
| 5272.649 | Hex_9_HexNAc_8_NeuAc_1_dHex_6_ |
| 5283.628 | Hex_7_HexNAc_6_NeuAc_4_dHex_5_ |
|  | Hex_10_HexNAc_10_NeuAc_2_ |
| 5285.644 | Hex_9_HexNAc_8_NeuAc_2_dHex_4_ |
| 5287.659 | Hex_11_HexNAc_10_dHex_3_ |
| 5298.639 | Hex_9_HexNAc_8_NeuAc_3_dHex_2_ |
| 5300.655 | Hex_11_HexNAc_10_NeuAc_1_dHex_1_ |
| 5313.675 | Hex_8_HexNAc_9_NeuAc_1_dHex_6_ |
| 5328.686 | Hex_10_HexNAc_11_dHex_3_ |
| 5341.67 | Hex_7_HexNAc_7_NeuAc_3_dHex_6_ |
| 5360.701 | Hex_10_HexNAc_9_dHex_6_ |
| 5371.681 | Hex_8_HexNAc_7_NeuAc_3_dHex_5_ |
| 5373.696 | Hex_10_HexNAc_9_NeuAc_1_dHex_4_ |
| 5388.707 | Hex_12_HexNAc_11_dHex_1_ |
| 5399.687 | Hex_10_HexNAc_9_NeuAc_3_ |
| 5461.749 | Hex_11_HexNAc_10_dHex_4_ |
| 5474.744 | Hex_11_HexNAc_10_NeuAc_1_dHex_2_ |
| 5487.739 | Hex_11_HexNAc_10_NeuAc_2_ |
| 5534.79 | Hex_10_HexNAc_9_dHex_7_ |
| 5545.781 | Hex_11_HexNAc_11_NeuAc_1_dHex_1_ |
| 5547.785 | Hex_10_HexNAc_9_NeuAc_1_dHex_5_ |
| 5562.796 | Hex_12_HexNAc_11_dHex_2_ |
| 5605.827 | Hex_10_HexNAc_10_dHex_6_ |
| 5635.838 | Hex_11_HexNAc_10_dHex_5_ |
| 5648.833 | Hex_11_HexNAc_10_NeuAc_1_dHex_3_ |
| 5661.828 | Hex_11_HexNAc_10_NeuAc_2_dHex_1_ |
| 5719.859 | Hex_8_HexNAc_7_NeuAc_3_dHex_7_ |
|  | Hex_11_HexNAc_11_NeuAc_1_dHex_2_ |
| 5721.875 | Hex_10_HexNAc_9_NeuAc_1_dHex_6_ |
| 5732.854 | Hex_8_HexNAc_7_NeuAc_4_dHex_5_ |
| 5732.865 | Hex_11_HexNAc_11_NeuAc_2_ |
| 5736.886 | Hex_12_HexNAc_11_dHex_3_ |
| 5749.881 | Hex_12_HexNAc_11_NeuAc_1_dHex_1_ |
| 5779.917 | Hex_10_HexNAc_10_dHex_7_ |
| 5809.927 | Hex_11_HexNAc_10_dHex_6_ |
| 5820.907 | Hex_9_HexNAc_8_NeuAc_3_dHex_5_ |
| 5822.922 | Hex_11_HexNAc_10_NeuAc_1_dHex_4_ |
| 5835.918 | Hex_11_HexNAc_10_NeuAc_2_dHex_2_ |
| 5895.964 | Hex_10_HexNAc_9_NeuAc_1_dHex_7_ |
| 5906.943 | Hex_8_HexNAc_7_NeuAc_4_dHex_6_ |
|  | Hex_11_HexNAc_11_NeuAc_2_dHex_1_ |
| 5910.975 | Hex_12_HexNAc_11_dHex_4_ |
| 5921.954 | Hex_10_HexNAc_9_NeuAc_3_dHex_3_ |
| 5923.97 | Hex_12_HexNAc_11_NeuAc_1_dHex_2_ |
| 5994.996 | Hex_9_HexNAc_8_NeuAc_3_dHex_6_ |
| 5997.012 | Hex_11_HexNAc_10_NeuAc_1_dHex_5_ |
| 6085.064 | Hex_12_HexNAc_11_dHex_5_ |
| 6098.059 | Hex_12_HexNAc_11_NeuAc_1_dHex_3_ |
| 6171.101 | Hex_11_HexNAc_10_NeuAc_1_dHex_6_ |
| 6259.153 | Hex_12_HexNAc_11_dHex_6_ |

Supplementary Table S2. Monosaccharide composition of CVF N-glycans with m/z values between 5000 and 7000 Da. CVF N-glycans were released by PNGase F, permethylated, and subsequently subjected to Sep-Pak cleanup ("Materials and Methods"). Data were acquired in the positive ion mode [M+Na]+. A 0.8 S/N threshold for peak detection was applied. Peak assignments are based on 12C isotopic composition together with knowledge of the biosynthetic pathways.
